# Association of the TyG index and its body fat distribution composites with CHD and MACE risk in adults with OSAHS: incremental discrimination and exploratory mediation analysis

**DOI:** 10.3389/fendo.2026.1848218

**Published:** 2026-06-16

**Authors:** Zeyu Liang, Xiaoli Zhu, Yulan Chen, Ayiguzaili Maimaitimin, Nayiman Nihemaiti, Gulixian Shata, Rehanguli Maihemutijiang

**Affiliations:** 1 Department of Hypertension, The First Affiliated Hospital of Xinjiang Medical University, Urumqi, China; 2 The Fourth Department of Cardiology, Xinjiang Medical University College of Traditional Chinese Medicine, Urumqi, China

**Keywords:** coronary heart disease, exploratory mediation analysis, insulin resistance, major adverse cardiovascular events, obstructive sleep apnea-hypopnea syndrome, TyG-related indices

## Abstract

**Introduction:**

Obstructive sleep apnea-hypopnea syndrome (OSAHS) is closely associated with coronary heart disease (CHD) and adverse cardiovascular outcomes, but the comparative value of the triglyceride-glucose (TyG) index and its body fat distribution composites for cardiovascular risk stratification in OSAHS remains unclear.

**Methods:**

We assessed the associations of TyG, TyG-weight-adjusted waist index (TyG-WWI), TyG-Chinese visceral adiposity index (TyG-CVAI), and TyG-body roundness index (TyG-BRI) with prevalent CHD and incident major adverse cardiovascular events (MACE) in adults with OSAHS. This single-center retrospective observational study included 1,122 adults with OSAHS who underwent both polysomnography and coronary angiography, including 533 patients with CHD and 589 without CHD. TyG-related indices were standardized and analyzed per 1-standard deviation increase. Multivariable logistic regression, restricted cubic spline analysis, Cox proportional hazards models, time-dependent receiver operating characteristic analysis, and exploratory statistical decomposition were performed.

**Results:**

All TyG-related indices were independently associated with prevalent CHD after full adjustment. TyG-WWI showed the strongest association (odds ratio 3.21, 95% confidence interval 2.68-3.89 per 1-standard deviation increase). Adding TyG-WWI to conventional risk factors plus apnea-hypopnea index provided the greatest improvement in CHD discrimination (area under the curve 0.802; integrated discrimination improvement 0.150; continuous net reclassification improvement 0.706; all P<0.001). During a median follow-up of 17 months, 80 MACE occurred, and TyG-WWI showed the strongest association with incident MACE among the evaluated indices(hazard ratio 1.545, 95% confidence interval 1.217-1.961). Exploratory statistical decomposition suggested potential indirect pathway signals involving TyG-WWI and TyG-CVAI.

**Discussion:**

In this selected high-risk cohort of adults with OSAHS undergoing coronary evaluation, TyG and its adiposity-based composites were associated with prevalent CHD and showed supportive associations with follow-up MACE. TyG-WWI showed the most consistent cross-sectional, supportive longitudinal, and incremental discriminative value, suggesting its potential as a candidate marker for cardiovascular risk stratification. Given the limited MACE events and the exploratory nature of the statistical decomposition, these findings should be interpreted cautiously and validated prospectively.

## 1 Introduction

Obstructive sleep apnea-hypopnea syndrome (OSAHS) is a prevalent sleep-disordered breathing condition characterized by repetitive upper airway collapse during sleep. These episodes result in apnea or hypopnea, which subsequently trigger intermittent hypoxia, sleep fragmentation, and fluctuations in intrathoracic negative pressure (1) Robust evidence has established that OSAHS is closely linked to various cardiovascular diseases, notably showing a significant association with the pathogenesis and progression of coronary heart disease (CHD) (2). The mechanisms by which OSAHS promotes cardiovascular injury are multifactorial, involving sustained sympathetic activation, heightened oxidative stress, inflammatory responses, endothelial dysfunction, and metabolic derangements (3).Notably, metabolic-adiposity phenotypic abnormalities—such as insulin resistance (IR), dyslipidemia, and central obesity or visceral fat accumulation—may further exacerbate OSAHS-related cardiovascular risk. Consequently, identifying high-risk individuals within the OSAHS population through the integrated assessment of metabolic dysfunction and body fat distribution is of profound clinical significance.

Given the potential role of these metabolic-adiposity phenotypes in OSAHS-related risk, insulin resistance has gained increasing attention as a core component of metabolic dysfunction. IR is defined by reduced peripheral tissue responsiveness to insulin and significantly elevates the risk of cardiovascular disease. The triglyceride-glucose (TyG) index, derived from fasting triglycerides (TG) and fasting plasma glucose (FPG), has emerged as a validated and cost-effective proxy for IR (4). Previous studies have demonstrated that elevated TyG levels are independently associated with an increased risk of CHD, greater anatomical severity of coronary atherosclerosis, and other adverse cardiovascular outcomes (4, 5). Furthermore, composite indices that integrate the TyG index with anthropometric measures—such as body mass index (BMI), waist circumference (WC), and waist-to-height ratio—have shown superior predictive performance for cardiometabolic risk compared to the TyG index alone (6, 7). Recently, novel TyG-related indices, including TyG-BRI (Body Roundness Index), TyG-WWI (Weight-adjusted Waist Index), and TyG-CVAI (Chinese Visceral Adiposity Index), have also been linked to various adverse outcomes, such as stroke, periodontitis, anxiety, and metabolic dysfunction-associated steatotic liver disease (MASLD) (8). By consolidating information on glucose-lipid metabolism and body fat distribution, these indices enhance the assessment of visceral adiposity and may help elucidate the metabolic pathways underlying OSAHS-related cardiovascular risk.

Against this background, a growing body of epidemiological evidence further indicates that OSAHS severity—typically quantified by the apnea-hypopnea index (AHI)—is associated with an increased risk of CHD and subsequent major adverse cardiovascular events (MACE), particularly in high-risk populations such as those with acute coronary syndrome (9, 10). Within the OSAHS population, the TyG index has been validated as a simple IR surrogate associated with cardiovascular risk assessment, increased CHD risk, and coronary atherosclerosis severity, suggesting its potential to supplement traditional risk factors in identifying high-risk subgroups (4, 11). Compared to BMI alone, incorporating fat distribution or central obesity data into metabolic indices is expected to more comprehensively characterize the composite phenotype associated with insulin resistance, visceral fat accumulation, and cardiometabolic injury. Nevertheless, previous research has largely focused on general populations, standalone OSAHS cohorts, or acute coronary syndrome settings. There remains a lack of systematic comparisons between the TyG index and its various body fat distribution composites in assessing both CHD and MACE risk specifically among OSAHS patients undergoing coronary evaluation, particularly those with CHD status confirmed via coronary angiography (12).

Despite existing evidence linking TyG-related metabolic indices to OSAHS-related risk, significant evidence gaps remain regarding the synergistic effects and clinical implications of OSAHS severity and TyG-related indices on CHD and MACE risk. Current studies often investigate the relationship between the TyG index and cardiovascular risk in isolation or within non-OSAHS populations. Even when research within OSAHS cohorts suggests correlations between TyG and CHD risk across different AHI strata, systematic evaluations of the interaction between sleep-breathing parameters and composite body fat distribution indices remain insufficient (11, 13). Moreover, while the TyG index is associated with higher cardiovascular risk in OSAHS patients, its incremental discriminative value—beyond traditional risk factors and OSAHS parameters—has not been fully validated in patients concurrently undergoing polysomnography and coronary evaluation (2, 11). Furthermore, although TyG composites incorporating central obesity have been linked to OSAHS risk and cardiovascular mortality, whether indices such as TyG-WWI, TyG-CVAI, and TyG-BRI participate in potential mediating pathways between OSAHS severity and subsequent MACE has yet to be systematically evaluated using a causal inference framework (14).

The primary objective of this study is to evaluate the associations between the TyG index and its body fat distribution composites (TyG-WWI, TyG-CVAI, and TyG-BRI) with the prevalence of CHD and the incidence of MACE in adult patients with OSAHS. Additionally, we aim to assess their incremental discriminative capacity—quantified by improvements in AUC, Net Reclassification Improvement (NRI), and Integrated Discrimination Improvement (IDI)—relative to traditional risk factors and AHI. Finally, we utilized a counterfactual framework to conduct an exploratory statistical decomposition of the AHI–MACE association through these indices. By better capturing the interplay between metabolism and obesity, these indices may provide complementary information for cardiovascular risk stratification in this high-risk population.

## 2 Materials and methods

### 2.1 Study population

This study is a single-center, retrospective observational study consisting of both cross-sectional and follow-up cohort analyses. We consecutively recruited patients admitted to the First Affiliated Hospital of Xinjiang Medical University between March 2020 and December 2024 who underwent both overnight polysomnography (PSG) and coronary angiography (CAG). Because all participants underwent clinically indicated CAG, the study cohort represents a selected hospitalized OSAHS population referred for coronary evaluation rather than a community-based OSAHS sample. Therefore, the study design may be subject to referral and indication bias. After excluding cases lost to follow-up, a total of 1,122 patients were included in the final analysis. The cohort comprised 533 patients with comorbid obstructive sleep apnea-hypopnea syndrome (OSAHS) and coronary heart disease (CHD), and 589 patients with OSAHS alone. A total of 80 major adverse cardiovascular events (MACE) were recorded during the study period.

### 2.2 Inclusion and exclusion criteria

#### 2.2.1 Inclusion criteria

①Age 18–80 years. ②OSAHS diagnosis based on clinical symptoms/signs combined with PSG results, following adult diagnostic recommendations; patients with an apnea-hypopnea index (AHI) ≥ 5 events/h were included (15).③CHD defined according to relevant guidelines (16), with the operational diagnostic criterion being CAG-confirmed stenosis of ≥ 50% in at least one major coronary artery (17).

#### 2.2.2 Exclusion criteria

Participants were excluded if they presented with central sleep apnea; severe cardiac, hepatic, or renal insufficiency; or malignancies. Further exclusions included chronic respiratory diseases, pulmonary or systemic infections; endocrine disorders such as hyperthyroidism, hypothyroidism, or acromegaly; primary systemic vasculitis; psychiatric disorders; or current use of sedative-hypnotic medications. This study was approved by the Medical Ethics Committee of the First Affiliated Hospital of Xinjiang Medical University (20200318-109).

### 2.3 Clinical data collection

General clinical data included sex, age, snoring status, comorbidities (e.g., hypertension, diabetes), smoking history, family history, and body mass index (BMI).

Laboratory Data: Fasting blood samples were collected the morning following an 8-hour fast. Parameters were measured using a Roche C8000 modular analyzer (Germany) and included blood urea nitrogen (BUN), serum creatinine (Scr), uric acid (UA), triglycerides (TG), total cholesterol (TC), high-density lipoprotein cholesterol (HDL-C), low-density lipoprotein cholesterol (LDL-C), alanine aminotransferase (ALT), aspartate aminotransferase (AST), and fasting plasma glucose (FPG).

Blood Pressure: 24-hour ambulatory blood pressure monitoring was performed using a Welch Allyn 6100 monitor (USA) to record 24-hour systolic (24hSBP) and diastolic (24hDBP) blood pressure.

### 2.4 Coronary angiography

Data were collected from suspected CHD patients indicated for CAG based on clinical criteria (e.g., chest pain, positive non-invasive tests, coronary CTA findings, or high-risk assessment). Procedures were performed using a GE Innova 2100 imaging system with continuous monitoring of ECG, blood pressure, and oxygen saturation. Selective CAG was conducted via the radial or femoral artery using the Seldinger technique and Judkins method. Images were interpreted by three cardiologists blinded to the patients’ PSG data.

Selection and referral considerations: Because CAG was performed according to clinical indications, inclusion in the present cohort was not random. Patients who completed both PSG and CAG may have had a higher pre-test probability of CHD and a greater cardiometabolic burden than general OSAHS patients. This may introduce referral, indication, and potential collider bias. Since detailed referral-pathway variables and quantitative CAG-indication scores were unavailable, these biases could not be fully adjusted for in the statistical models.

### 2.5 Polysomnography

Overnight PSG was performed using a Compumedics system (Australia). Post-monitoring data were analyzed using Remlogic software and manually reviewed by professional technicians. Key metrics recorded included AHI, mean oxygen saturation (MSaO_2_), and lowest oxygen saturation (LSaO_2_).

### 2.6 Index calculation

The TyG index and its body fat distribution composites integrate metabolic data with anthropometric parameters to enhance the assessment of metabolic dysfunction and visceral adiposity. Since the original definition of the TyG index uses units of mg/dL for TG and FPG, mmol/L values were converted accordingly (TG × 88.57; FPG × 18) (8, 18, 19). Calculations were performed as follows:

TyG Index: TyG=ln[TG(mg/dL)×FPG(mg/dL)/2]Composite Indices: TyG-BRI = TyG × BRI; TyG-WWI = TyG × WWI; TyG-CVAI = TyG × CVAI;


$$BRI=364.2-365.5\times \sqrt{1-{\left(WC\;\left(cm\right)/2\pi \right)}^{2}/{\left(height\;\left(cm\right)/2\right)}^{2}}$$

$$WWI=WC\left(cm\right)/\sqrt{weight\left(kg\right)}$$

$$\begin{array}{rr} Male:CVAI & =-267.93+0.68\times age\;\left(years\right)+0.03\times BMI\;\left(kg/m^{2}\right)\\ +4.00\times WC\;\left(cm\right)+22.00\times \log_{10}(TG)\;\left(mmol/L\right)\\ -16.32\times HDL-C\left(mmol/L\right) \end{array}$$

$$\begin{array}{rr} Female:CVAI & =-187.32+1.71\times age\;\left(years\right)+4.23\times BMI\;\left(kg/m^{2}\right)\\ +1.12\times WC\;\left(cm\right)+39.76\times \log_{10}(TG)\;\left(mmol/L\right)\\ -11.66\times HDL-C\;\left(mmol/L\right) \end{array}$$

### 2.7 Major adverse cardiovascular events

In consideration of the established influence of OSAHS on cardiac rhythm and hemodynamics, this study adopted an expanded definition of MACE. This comprehensive composite endpoint includes cardiac death, non-fatal myocardial infarction (MI), stroke, arrhythmia, revascularization, and hospitalization for heart failure (10). The follow-up baseline was defined as the date of hospital discharge following the completion of both polysomnography (PSG) and coronary angiography (CAG), or whichever occurred later. The follow-up endpoint was established as the time to the first documented occurrence of a MACE or the date of the final follow-up.

Acknowledging the potential heterogeneity inherent in composite endpoints, the specific distribution of constituent events is detailed in the Results section, with their potential clinical implications addressed in the Discussion. Furthermore, “Hard MACE”—defined as a composite of cardiac death, non-fatal MI, stroke, and revascularization—was utilized as a sensitivity endpoint to verify the robustness of the primary conclusions against varying endpoint definitions (20).

### 2.8 Statistical analysis

#### 2.8.1 General analysis

For continuous variables, normality was assessed using the Kolmogorov-Smirnov test. Normally distributed data are presented as mean ± standard deviation (SD) and compared using Student’s t-test; non-normally distributed data are expressed as median (interquartile range, IQR) and compared using the Mann–Whitney U test. Categorical variables are reported as frequencies (percentages) and compared using the χ^2^test or Fisher’s exact test. During the data cleaning and cohort construction phase, the integrity of baseline variables was verified. Individuals with missing key baseline data (required for calculating primary indices or core covariates) were excluded prior to enrollment. For longitudinal analysis, individuals lost to follow-up or lacking outcome information were further excluded. These procedures ensured that the final analytical dataset consisted of complete-case data.

The primary exposures were the TyG index and its body fat distribution composites (TyG-WWI, TyG-CVAI, TyG-BRI). Except for baseline descriptive statistics, these exposures were Z-standardized before entering inferential models to allow the effect sizes to be interpreted as the risk change per 1-standard deviation (1−SD) increase. Spearman correlation coefficients were used to evaluate the interrelationships between TyG and its composites. To mitigate the risk of multicollinearity and overfitting, we employed the Boruta algorithms an exploratory, data-driven approach for feature selection of potential CHD/MACE predictors. To avoid redundancy, the primary exposures, their constituent components, and related variables were excluded from the Boruta process. It should be noted that Boruta results served only as exploratory/sensitivity references; the primary analysis relied on progressively adjusted models with pre-specified covariates. Multicollinearity among retained continuous covariates was assessed using the Variance Inflation Factor (VIF) and Spearman correlation matrices; variables with a VIF<5were included in the final models. Given the high correlation between AHI and oxygen desaturation metrics (MSaO_2_ and LSaO_2_), AHI was prioritized in the main models to avoid collinearity, while oxygen metrics were used in sensitivity analyses. Similarly, LDL-C was prioritized over total cholesterol (TC). Although ALT showed importance in feature selection, it was reserved for sensitivity analysis to avoid over-adjustment, as it likely resides within the metabolic dysfunction pathway.

#### 2.8.2 Cross-sectional analysis

Multivariable logistic regression was used to evaluate the association between TyG-related indices and CHD prevalence, with results reported as odds ratios (OR) and 95% confidence intervals (CI). Exposures entered the models as continuous variables (Z-standardized) to estimate the OR per 1−SD increase. Additionally, exposures were categorized into quartiles, with the median of each quartile treated as a continuous variable to calculate the P-for-trend. A progressive adjustment strategy was employed: Model 1 was unadjusted; Model 2 adjusted for traditional risk factors (age, sex, hypertension, diabetes, systolic blood pressure, smoking, LDL-C, and WBC count); and Model 3 further adjusted for AHI.

#### 2.8.3 Model performance and validation

To visualize the gradient relationship between Z-standardized TyG indices and CHD prevalence, we calculated the crude prevalence (%) across quartiles (Q1–Q4). Restricted cubic splines (RCS) with three knots (at the 10th, 50th, and 90th percentiles) were integrated into the multivariable logistic framework to examine dose-response relationships and potential non-linearity, using Z = 0 as the reference (OR = 1). Overall association (P_overall_) and non-linearity (P_non−linearity_) were assessed via Wald tests and likelihood ratio tests comparing the spline model against the linear model. To ensure stability, RCS curves are displayed between the 5th and 95th percentiles. If non-linearity was detected (P < 0.05), two-piecewise logistic regression was performed. The inflection point was estimated using the maximum likelihood method, and ORs were reported for segments above and below the threshold.

Predictive performance was evaluated by adding AHI to a baseline model of traditional risk factors, followed by the incremental addition of each Z-standardized TyG-related index. Discrimination was assessed via ROC curves and AUC, with differences compared using the DeLong test. Incremental utility was quantified using Integrated Discrimination Improvement (IDI) and continuous Net Reclassification Improvement (NRI). Internal validation involved bootstrap resampling (B = 1000) and repeated stratified 10-fold cross-validation. Calibration was assessed via out-of-fold (OOF) calibration plots and Brier scores, while clinical utility was examined through Decision Curve Analysis (DCA). Subgroup analyses utilized interaction terms to test for effect modification.

#### 2.8.4 Longitudinal analysis

Cox proportional hazards models were used to assess the association between TyG-related indices and MACE risk, reporting hazard ratios (HR) and 95% CI. The proportional hazards assumption was verified using Schoenfeld residuals. Kaplan–Meier curves and log-rank tests were used to visualize cumulative incidence across quartiles. For temporal stability, time-dependent ROC analysis with Inverse Probability of Censoring Weighting (IPCW) provided dynamic AUCs at 12, 18, and 24 months. Sensitivity analyses included evaluating “Hard MACE” and employing L2-penalized (Ridge) Cox regression to ensure robustness given the limited number of events (low Events Per Variable, EPV). The penalty parameter λ was optimized via 5-fold cross-validation based on Harrell’s C-index.

#### 2.8.5 Exploratory statistical decomposition

To enhance comparability and numerical stability, AHI was Z-standardized (ZAHI)exclusively for this section. We performed an exploratory counterfactual-based statistical decomposition to estimate the total effect (TE), natural direct effect (NDE), and natural indirect effect (NIE) of Z-standardized AHI on MACE through each Z-standardized TyG-related index as a candidate mediator. Empirical 95% CIs were obtained using 5,000 bootstrap iterations. Because AHI and candidate mediators were measured concurrently at baseline, temporal ordering could not be confirmed; therefore, this analysis was interpreted as hypothesis-generating rather than evidence of causal mediation.

All statistical tests were two-sided, and a P-value < 0.05 was considered statistically significant.

## 3 Results

### 3.1 Comparison of baseline characteristics between OSAHS and OSAHS-CHD groups

A total of 1,122 patients with OSAHS were enrolled and stratified into the CHD group (n=533) and the non-CHD group (n=589) based on coronary angiography findings (**Table 1**). While the age difference between the groups did not reach statistical significance (51 vs. 52 years, P = 0.058), the CHD group exhibited a significantly higher proportion of male participants (87.2% vs. 78.6%, P<0.001). Regarding sleep-disordered breathing parameters, the CHD group presented with significantly higher AHI and lower LSaO_2_ (both P<0.001). Additionally, the prevalence of hypertension, diabetes mellitus, and smoking history was markedly higher in the CHD group (all P<0.001).

**Table 1:** Baseline characteristics of patients with OSAHS and those with comorbid OSAHS and CHD.

Characteristic	OSAHS (n = 589)	OSAHS + CHD (n = 533)	P value
Demographics.			
Age, years	51 (44, 57)	52 (45, 59)	0.058
Male, n (%)	463 (78.6)	465 (87.2)	<0.001
Female, n (%)	126 (21.4)	68 (12.8)	
Sleep-related indices			
MSaO_2_, %	92.1 (91.2, 93.0)	92.1 (91.0, 93.0)	0.251
LSaO_2_, %	81.0 (76.75, 85.0)	80.0 (75.0, 84.0)	<0.001
AHI, events/h	21.5 (12.6, 35.5)	28.0 (18.6, 42.33)	<0.001
Blood pressure			
24-h SBP, mmHg	127 (118, 136)	130 (120, 142)	<0.001
24-h DBP, mmHg	81 (74, 89)	82 (76, 90)	0.011
Medical history			
Hypertension, n (%)	352 (59.8)	384 (72.0)	<0.001
Diabetes mellitus, n (%)	58 (9.8)	106 (19.9)	<0.001
Lifestyle factors			
Current smoking, n (%)	296 (50.3)	335 (62.9)	<0.001
Current drinking, n (%)	231 (39.2)	230 (43.2)	0.181
Laboratory indices			
WBC, ×10^9^/L	6.47 (5.39, 7.85)	7.04 (5.90, 8.26)	<0.001
PLT, ×10^9^/L	217 (183, 254)	221 (188, 264)	0.052
ALT, U/L	24.2 (16.83, 34.28)	24.65 (19.1, 36.1)	0.026
AST, U/L	20.2 (16.9, 25.6)	21.5 (17.6, 27.4)	0.007
Scr, μmol/L	72.0 (62.0, 82.4)	75.0 (65.97, 85.0)	<0.001
BUN, mmol/L	5.2 (4.4, 6.3)	5.5 (4.6, 6.5)	0.012
Lipid and glucose profile			
TC, mmol/L	4.26 (3.65, 4.90)	4.47 (3.94, 5.15)	<0.001
LDL-C, mmol/L	2.73 (2.22, 3.27)	2.93 (2.44, 3.40)	<0.001
HDL-C, mmol/L	1.07 (0.93, 1.29)	0.96 (0.83, 1.10)	<0.001
TG, mmol/L	1.58 (1.13, 2.24)	2.02 (1.57, 2.80)	<0.001
FPG, mmol/L	5.01 (4.57, 5.68)	5.27 (4.78, 6.18)	<0.001
Anthropometric indices			
BMI, kg/m²	27.12 (24.44, 30.02)	27.76 (25.25, 30.80)	0.003
WC, cm	87.9 (82.6, 93.9)	93.2 (87.1, 99.5)	<0.001
TyG-related indices			
TyG	8.75 (8.40, 9.18)	9.11 (8.80, 9.48)	<0.001
TyG-BRI	32.47 (26.09, 38.88)	39.01 (31.36, 47.02)	<0.001
TyG-WWI	86.74 (82.58, 92.02)	94.60 (89.73, 99.63)	<0.001
TyG-CVAI	965.77 (765.20, 1188.70)	1202.40 (996.39, 1463.40)	<0.001

**Table 1**. Data are presented as median (IQR) for continuous variables and n (%) for categorical variables. Between-group comparisons were performed using the Student’s t-test or Mann–Whitney U test for continuous variables, and the chi-square test or Fisher’s exact test for categorical variables, as appropriate. In this table, the TyG index and its composite indices (TyG-CVAI, TyG-WWI, and TyG-BRI) are presented on their original scales to describe the baseline distribution of the study population; in all subsequent inferential analyses, these exposure variables were Z-standardized before model fitting.

In terms of laboratory and metabolic profiles, the CHD group showed significantly higher levels of WBC, TC, LDL-C, TG, and FPG, alongside lower HDL-C (all P<0.001). Correspondingly, the TyG index and its body fat distribution composites (TyG-BRI, TyG-WWI, and TyG-CVAI) were significantly elevated in patients with comorbid CHD compared to those with OSAHS alone (all P<0.001). Spearman correlation analysis (**Supplementary Figure 1**) indicated moderate correlations between the TyG index and its composites (r=0.32–0.74). Specifically, the TyG index showed the strongest correlation with TyG-WWI (r=0.74), followed by TyG-CVAI (r=0.46) and TyG-BRI (r=0.32), suggesting that while these indices are inter-related, they are not entirely redundant and capture distinct aspects of metabolic and adiposity dysfunction.

Abbreviations: OSAHS, obstructive sleep apnea–hypopnea syndrome; CHD, coronary heart disease; 24-h SBP, 24-hour systolic blood pressure; 24-h DBP, 24-hour diastolic blood pressure; AHI, apnea–hypopnea index; MSaO_2_, mean oxygen saturation; LSaO_2_, lowest oxygen saturation; BMI, body mass index; WC, waist circumference; WBC, white blood cell count; PLT, platelet count; ALT, alanine aminotransferase; AST, aspartate aminotransferase; Scr, serum creatinine; BUN, blood urea nitrogen; TC, total cholesterol; LDL-C, low-density lipoprotein cholesterol; HDL-C, high-density lipoprotein cholesterol; TG, triglycerides; FPG, fasting plasma glucose; TyG, triglyceride–glucose index; TyG-BRI, triglyceride–glucose index–body roundness index; TyG-WWI, triglyceride–glucose index–weight-adjusted waist index; TyG-CVAI, triglyceride–glucose index–Chinese visceral adiposity index.

### 3.2 Correlation and multicollinearity assessment of TyG-related indices

**Figure 1:**
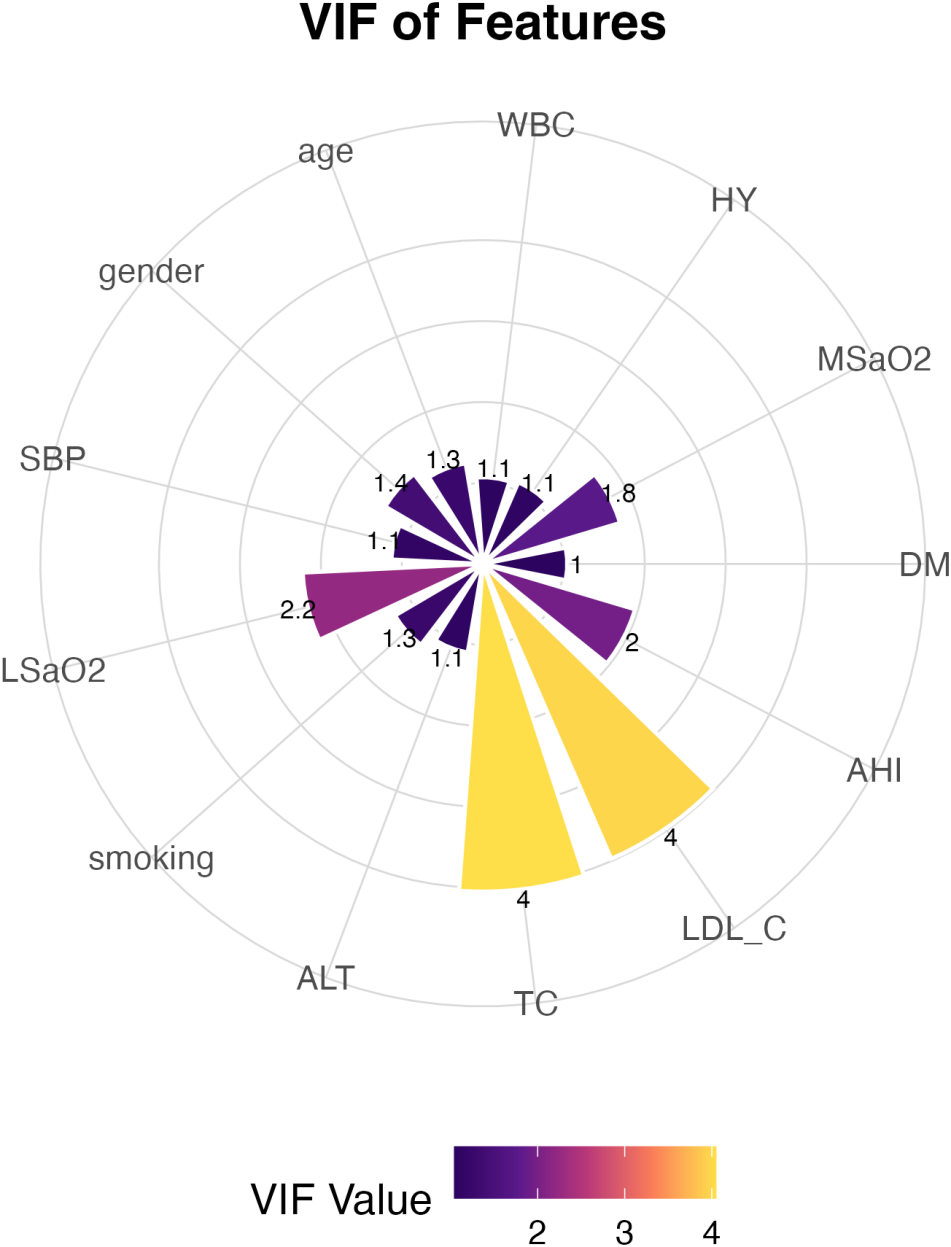
Variance inflation factors (VIFs) for candidate continuous covariates included in the multivariable models. All covariates retained in the primary models had VIF values<5, indicating an acceptable risk of multicollinearity.

**Figure 2:**
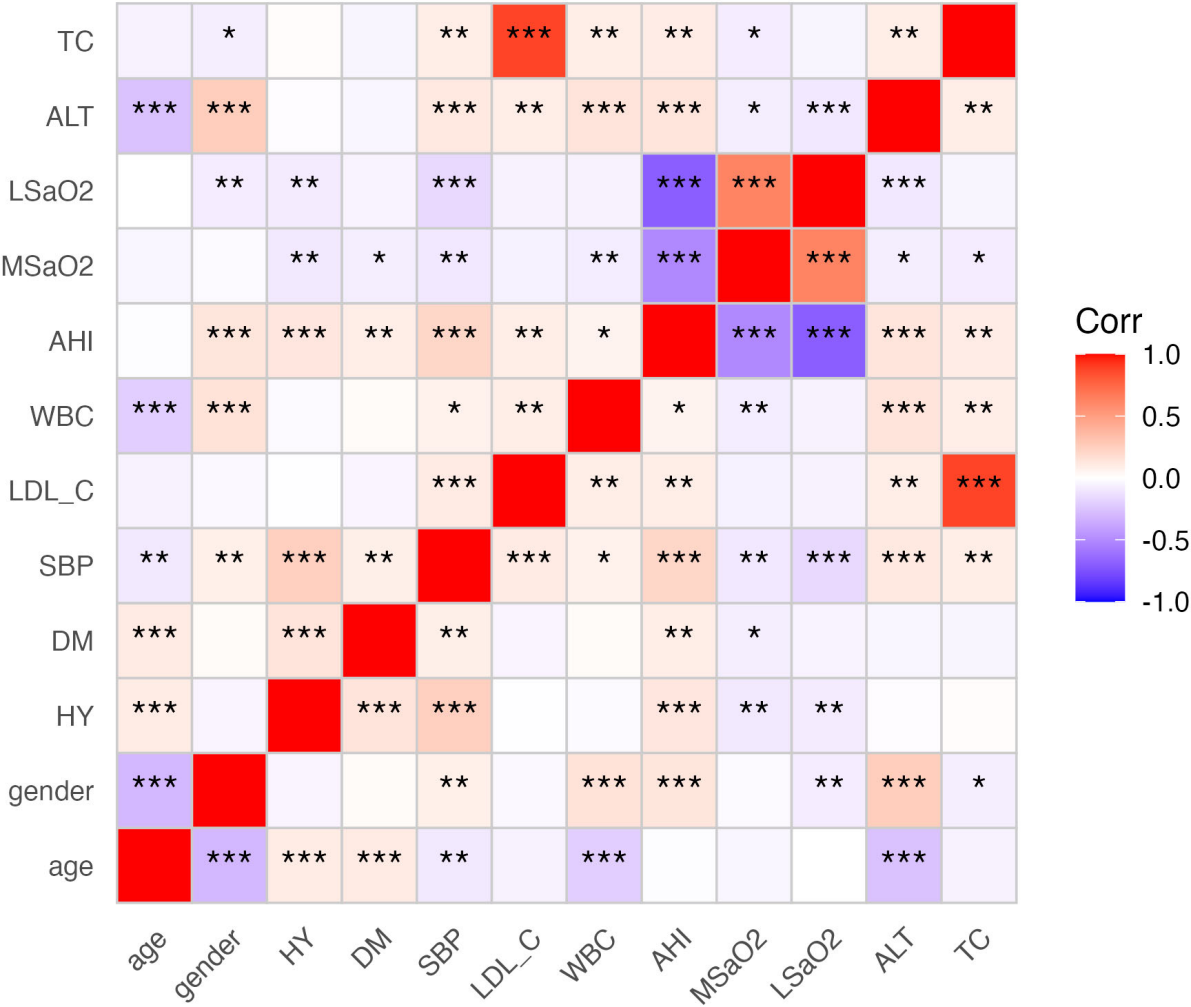
Spearman correlation matrix of candidate covariates. This heatmap illustrates the correlation coefficients between candidate variables. The intensity of the color corresponds to the magnitude of the correlation, while the color scale denotes the direction of the relationship (positive or negative); numerical values within the cells represent the respective correlation coefficients. This figure facilitates the assessment of the correlation structure and supplements the VIF results in identifying potential multicollinearity. *P < 0.05, **P < 0.01, and ***P < 0.001.

The Boruta algorithm was implemented as an exploratory reference for covariate selection (**Supplementary Figure 2**). To minimize the risk of multicollinearity, the Variance Inflation Factor (VIF) was calculated for the retained continuous covariates (**Figure 1**), and their inter-relationships were further evaluated using a Spearman correlation matrix (**Figure 2**). To prevent the redundant introduction of variables, the TyG index, its composite indices, and their individual constituent components were excluded from the candidate covariate pool. All covariates included in the final models exhibited VIF values<5, indicating that the risk of multicollinearity was statistically acceptable.

Given the high collinearity between the apnea-hypopnea index (AHI) and oxygen desaturation metrics (MSaO_2_ and LSaO_2_), AHI was prioritized for inclusion in the primary models; MSaO_2_ and LSaO_2_ were subsequently used in sensitivity analyses to verify the robustness of the findings. Similarly, due to the strong correlation between total cholesterol (TC) and LDL-C, LDL-C was preferentially included in the primary models, with TC serving as a substitute for validation in sensitivity analyses.

### 3.3 Association of TyG index and its composite indices with CHD

Although the Boruta algorithm suggested a degree of importance for alanine aminotransferase (ALT), it primarily reflects hepatocyte injury or a metabolic dysfunction-associated steatotic liver disease (MASLD) phenotype, which is susceptible to fluctuations from alcohol consumption, medication, or acute inflammation. Furthermore, since ALT likely resides within the potential “metabolic derangement → cardiovascular risk” pathway, its premature inclusion in the primary models could lead to over-adjustment and obscure the mechanistic interpretation. Consequently, ALT was excluded from the primary models as a mandatory covariate and was instead utilized as an extended covariate in sensitivity analyses to evaluate the robustness of our conclusions.

To assess the association between the TyG index and its body fat distribution composites with the risk of CHD, we constructed incrementally adjusted multivariable logistic regression models (**Table 2**). For the continuous variable analysis, all TyG-related indices were entered into the models in Z-standardized form to facilitate a direct comparison of effect sizes. All four indices were significantly associated with an increased risk of CHD, with TyG-WWI exhibiting the strongest association and remaining relatively stable across progressive adjustments. Each 1-SD increase in Z-standardized TyG-WWI was associated with a markedly elevated risk of CHD: Model 1 OR = 3.59 (95% CI 3.03–4.31, P < 0.001); Model 2 OR = 3.29 (95% CI 2.75–3.97, P < 0.001); and Model 3 OR = 3.21 (95% CI 2.68–3.89, P < 0.001).

**Table 2:** Logistic regression analysis of Z-standardized TyG-related indices and CHD risk.

Exposure/comparison	Model 1 OR (95% CI)	P value	Model 2 OR (95% CI)	P value	Model 3 OR (95% CI)	P value
TyG						
Per 1-SD increase	2.10 (1.84, 2.42)	<0.001	1.90 (1.64, 2.21)	<0.001	1.84 (1.59, 2.15)	<0.001
Q1	Reference		Reference		Reference	
Q2 vs Q1	2.70 (1.89, 3.90)	<0.001	2.30 (1.58, 3.37)	<0.001	2.30 (1.58, 3.39)	<0.001
Q3 vs Q1	4.54 (3.17, 6.56)	<0.001	3.47 (2.36, 5.13)	<0.001	3.41 (2.31, 5.06)	<0.001
Q4 vs Q1	5.54 (3.86, 8.04)	<0.001	4.18 (2.83, 6.23)	<0.001	3.93 (2.65, 5.88)	<0.001
P for trend		<0.001		<0.001		<0.001
TyG-WWI						
Per 1-SD increase	3.59 (3.03, 4.31)	<0.001	3.29 (2.75, 3.97)	<0.001	3.21 (2.68, 3.89)	<0.001
Q1	Reference		Reference		Reference	
Q2 vs Q1	5.77 (3.80, 8.98)	<0.001	5.11 (3.33, 8.03)	<0.001	5.07 (3.29, 7.97)	<0.001
Q3 vs Q1	9.14 (6.02, 14.22)	<0.001	7.47 (4.85, 11.75)	<0.001	7.42 (4.81, 11.71)	<0.001
Q4 vs Q1	25.66 (16.46, 41.05)	<0.001	20.19 (12.72, 32.84)	<0.001	18.84 (11.84, 30.72)	<0.001
P for trend		<0.001		<0.001		<0.001
TyG-BRI						
Per 1-SD increase	1.92 (1.67, 2.23)	<0.001	1.79 (1.53, 2.09)	<0.001	1.71 (1.47, 2.01)	<0.001
Q1	Reference		Reference		Reference	
Q2 vs Q1	1.67 (1.18, 2.37)	0.004	1.48 (1.02, 2.14)	0.039	1.49 (1.03, 2.16)	0.037
Q3 vs Q1	2.84 (2.01, 4.04)	<0.001	2.34 (1.62, 3.40)	<0.001	2.31 (1.60, 3.37)	<0.001
Q4 vs Q1	4.75 (3.34, 6.82)	<0.001	3.92 (2.66, 5.82)	<0.001	3.53 (2.38, 5.26)	<0.001
P for trend		<0.001		<0.001		<0.001
TyG-CVAI						
Per 1-SD increase	2.36 (2.04, 2.75)	<0.001	2.16 (1.85, 2.55)	<0.001	2.07 (1.76, 2.45)	<0.001
Q1	Reference		Reference		Reference	
Q2 vs Q1	2.09 (1.46, 3.01)	<0.001	1.84 (1.26, 2.69)	0.002	1.79 (1.23, 2.63)	0.003
Q3 vs Q1	3.77 (2.64, 5.43)	<0.001	3.27 (2.24, 4.80)	<0.001	3.07 (2.09, 4.52)	<0.001
Q4 vs Q1	7.73 (5.34, 11.33)	<0.001	6.03 (4.03, 9.11)	<0.001	5.38 (3.57, 8.19)	<0.001
P for trend		<0.001		<0.001		<0.001

**Table 2**.Multivariable logistic regression was used to analyze the associations of Z-standardized TyG, TyG-WWI, TyG-CVAI, and TyG-BRI with CHD risk. Exposure indices were modeled per 1-standard deviation (1-SD) increase and further analyzed by quartiles. Model 1: Unadjusted. Model 2: Adjusted for age, sex, hypertension, diabetes, systolic blood pressure, smoking status, LDL-C, and WBC count. Model 3: Further adjusted for AHI based on Model 2.P_for trend_: Calculated by including the median value of each quartile as a continuous variable in the model.

Analysis by quartile groups further revealed a clear dose-response relationship. Compared to the lowest quartile (Q1), the risk of CHD increased monotonically across higher TyG-WWI quartiles. In Model 3, the ORs for Q2, Q3, and Q4 were 5.07 (95% CI 3.29–7.97), 7.42 (95% CI 4.81–11.71), and 18.84 (95% CI 11.84–30.72), respectively, with a statistically significant trend (P_for trend_ < 0.001). Other TyG-related indices also showed significant associations with CHD risk, albeit with smaller effect sizes. In Model 3, the OR per 1-SD increase was 1.84 (95% CI 1.59–2.15,P < 0.001) for TyG; 2.07 (95% CI 1.76–2.45, P < 0.001) for TyG-CVAI; and 1.71 (95% CI 1.47–2.01, P < 0.001) for TyG-BRI. Their respective quartile analyses and trend tests yielded consistent results, confirming that these indices are independently associated with CHD.

Sensitivity analyses (**Supplementary Table 1**) demonstrated that the direction and significance of these associations remained generally consistent after additional adjustment for ALT, substituting TC for LDL-C, or replacing AHI with MSaO_2_ or LSaO_2_. Subgroup analyses (**Supplementary Figure 3**) further indicated that these associations were consistent across different strata of sex, age, hypertension, diabetes, smoking status, LDL-C levels, and AHI severity, with no significant interactions observed (all P_for interaction_>0.05).

### 3.4 Restricted cubic splines revealing non-linear dose-response relationships between TyG-related indices and CHD risk

**Figure 3:**
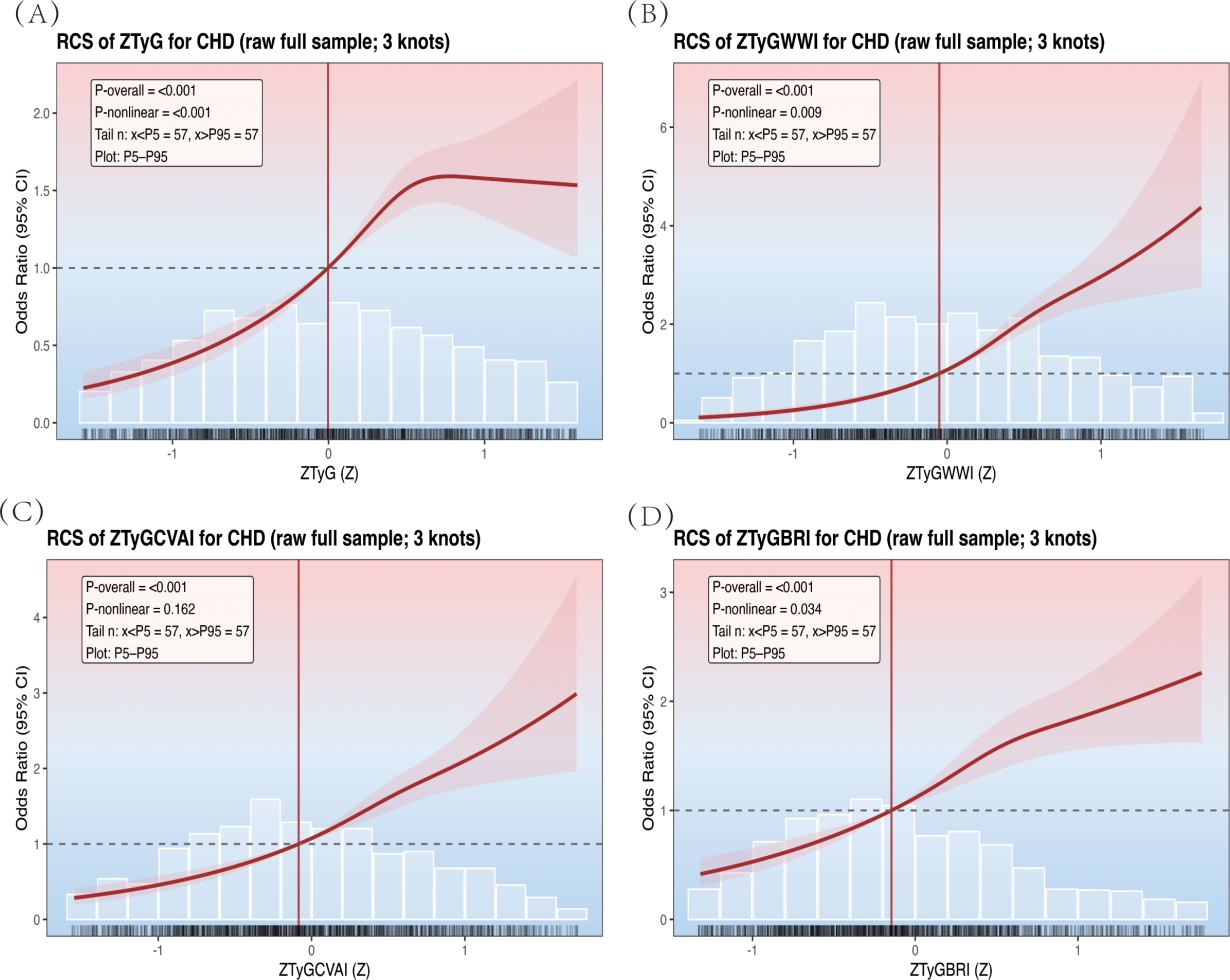
Dose-response relationships were evaluated in the fully adjusted logistic regression model (Model 3) for **(A)** ZTyG, **(B)** ZTyG-WWI, **(C)** ZTyG-CVAI, and **(D)** ZTyG-BRI. Splines were fitted with three knots at the 10th, 50th, and 90th percentiles, using Z = 0 as the reference (OR = 1). Solid lines represent the adjusted odds ratios (ORs), and shaded areas denote the 95% confidence intervals (CIs). Histograms illustrate the exposure distribution. To minimize instability from sparse extreme values, curves are displayed within the 5th–95th percentile range, with tail sample sizes (x<P5 and x>P95) reported. P_overall_ represents the joint test for the overall association, and P_non−linear_ tests for deviations from linearity.

In the fully adjusted multivariable logistic regression model (Model 3; adjusting for age, sex, hypertension, diabetes, systolic blood pressure, smoking history, LDL-C, WBC count, and AHI), restricted cubic spline (RCS) analysis demonstrated that all four TyG-related indices, when modeled as continuous variables, exhibited significant overall associations with CHD risk (all P_overall_<0.001; **Figure 3**). Specifically, when Z-standardized indices were incorporated, TyG (P_non−linear_<0.001), TyG-WWI (P_non−linear_=0.009), and TyG-BRI (P_non−linear_=0.034) showed significant non-linear associations with CHD risk. In contrast, no significant evidence of non-linearity was observed for TyG-CVAI (P_non−linear_=0.162). To mitigate the potential instability of the spline curves caused by sparse data at the extremes of the distribution, the fits are displayed only within the 5th to 95th percentile range, with 57 cases reported at each tail.

Furthermore, when stratified by quartiles based on the original distribution of the study sample, the crude prevalence of CHD exhibited a monotonic increasing trend from Q1 to Q4 for all indices (**Supplementary Figure 4**). Specifically, the prevalence rose from 23.5% (66/281) to 63.0% (177/281) for TyG, from 24.2% (68/281) to 71.2% (200/281) for TyG-CVAI, and from 29.2% (82/281) to 66.2% (186/281) for TyG-BRI. Notably, TyG-WWI exhibited the most pronounced gradient, with the crude prevalence of CHD increasing from 12.1% (34/281) in Q1 to 77.9% (219/281) in Q4.

To further evaluate potential threshold effects, we utilized two-piecewise logistic regression for the Z-standardized indices and compared the piecewise models to single linear models using the likelihood ratio test (LRT). Consistent with the RCS findings, inflection points were identified at Z=−1.089 for TyG, Z=−0.407 for TyG-WWI, and Z = 0.241 for TyG-BRI (**Table 3**). For TyG, a moderately strong positive association with CHD risk was observed above the inflection point (OR = 1.39 per 1-SD increase, 95% CI 1.17–1.66; n/events=975/522), whereas the estimate below the inflection point was less precise due to limited events (n/events=147/11). For TyG-WWI, significant positive associations were observed on both sides of the inflection point, with a markedly stronger correlation noted above the threshold (OR = 2.19 per 1-SD increase, 95% CI 1.67–2.87; n/events=721/457; LRT P < 0.0001). For TyG-BRI, the association was stronger below the inflection point (OR = 2.21 per 1-SD increase, 95% CI 1.55–3.16; n/events=745/292) and attenuated above the threshold while remaining statistically significant (OR = 1.36, 95% CI 1.02–1.81; n/events=377/241; LRT P = 0.0367).

**Table 3:** Threshold effects of Z-standardized TyG-related indices on CHD risk in the fully adjusted model.

Exposure (per1-SD)	P for nonlinearity	Inflection point (Z)	OR below inflection point (95% CI)	n/events below	OR above inflection point (95% CI)	n/events above	P for LRT
TyG	<0.001	−1.089	15940.21 (9.10, 2,793,458.63)*	147/11	1.39 (1.17, 1.66)	975/522	<0.001
TyGWWI	0.007	−0.407	12.58 (4.77, 33.16)	401/76	2.19 (1.67, 2.87)	721/457	<0.001
TyGBRI	0.034	0.241	2.21 (1.55, 3.16)	745/292	1.36 (1.02, 1.81)	377/241	0.0367

**Table 3**. Threshold effects were evaluated in the fully adjusted model (Model 3). Inflection points (expressed in Z-units) were estimated using the maximum likelihood method. Odds ratios (ORs) and 95% confidence intervals (CIs) per 1-SD increase are presented for segments above and below the inflection point, along with respective event counts. P-values for non-linearity were derived from the RCS terms in Model 3. The likelihood ratio test (LRT) was used to compare the two-piecewise model with the single linear model. *Estimates for ZTyG below the inflection point were unstable due to event sparsity (n/events=147/11) and should be interpreted with caution.

### 3.5 Incremental predictive performance of TyG-related indices for CHD

**Figure 4:**
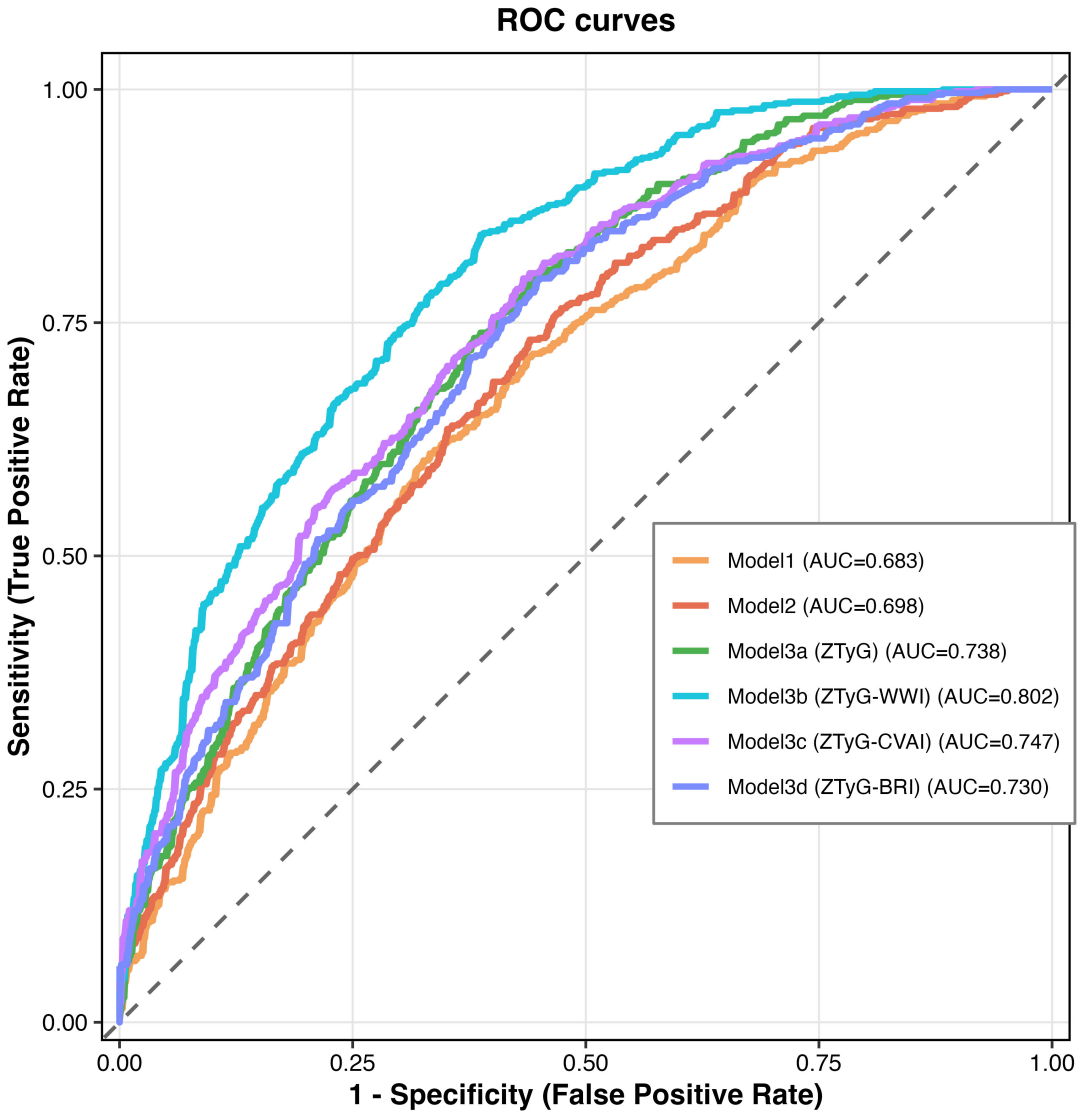
Receiver operating characteristic (ROC) curves compare the discriminative capacity of nested models for CHD. Model 1:Traditional risk factors (age, sex, hypertension, diabetes, 24hSBP, smoking, LDL-C, and WBC count); Model 2: Model 1 + raw-scale AHI; Models 3a–3d: Model 2 + Z-standardized TyG, TyG-WWI, TyG-CVAI, and TyG-BRI, respectively. The Area Under the Curve (AUC) was used to quantify discriminative performance.

ROC analysis revealed that the baseline model constructed from traditional risk factors (Model 1: age, sex, hypertension, diabetes, 24hSBP, smoking, LDL-C, and WBC count) yielded a discriminative capacity for CHD of AUC = 0.683 (**Figure 4**; **Table 4**). The subsequent addition of raw-scale AHI to Model 1 (Model 2) increased the AUC to 0.698 (ΔAUC=0.015, DeLong P = 0.033), with an IDI of 0.018 (95% CI 0.010–0.026) and a continuous NRI of 0.229 (95% CI 0.114–0.338). these results suggest that AHI provides modest but statistically significant incremental discriminative information beyond traditional risk factors.

**Table 4:** Incremental discrimination of progressive models for CHD.

Comparison	Reference model AUC	New model AUC	ΔAUC	DeLong P	IDI (95% CI)	Continuous NRI (95% CI)
Model1 → Model 2	0.683	0.698	0.015	0.033	0.018 (0.010–0.026)	0.229 (0.114–0.338)
Model2 → Model 3a (TyG)	0.698	0.738	0.040	<0.001	0.052 (0.039–0.065)	0.397 (0.282–0.513)
Model2 → Model 3b (TyG-WWI)	0.698	0.802	0.104	<0.001	0.150 (0.131–0.170)	0.706 (0.600–0.810)
Model2 → Model 3c (TyG-CVAI)	0.698	0.747	0.049	<0.001	0.065 (0.050–0.080)	0.500 (0.383–0.612)
Model2 → Model 3d (TyG-BRI)	0.698	0.730	0.032	<0.001	0.039 (0.027–0.051)	0.419 (0.303–0.535)

**Table 4**. This table summarizes the incremental improvements in model performance, including AUC, ΔAUC, DeLong P-values, IDI, and continuous NRI. Model definitions are consistent with **Figure 4**. ΔAUC represents the difference in AUC between the new model and the preceding reference model. IDI and continuous NRI quantify improvements in incremental discrimination and reclassification, respectively.

**Figure 5:**
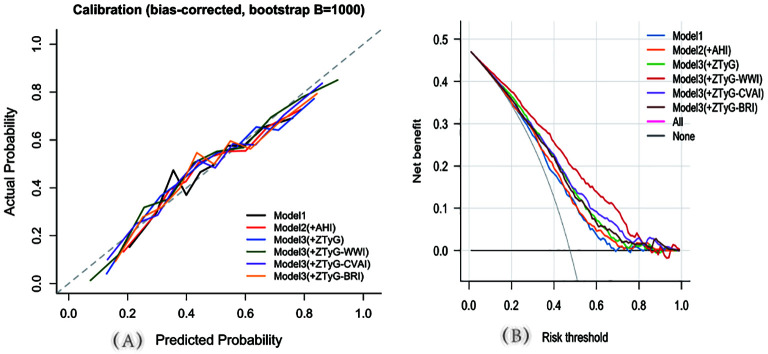
Supportive validation results for the predictive models. **(A)** Calibration Curves: Evaluates model calibration by comparing predicted probabilities with actual observed risks; the 45° dashed line represents ideal calibration. **(B)** Decision Curve Analysis (DCA) Evaluates the clinical net benefit of different models across various threshold probabilities. “Treat all” and “Treat none” serve as reference strategies. A higher curve indicates greater potential clinical utility within the corresponding threshold range.

Building upon Model 2, the independent inclusion of TyG, TyG-WWI, TyG-CVAI, or TyG-BRI as Z-standardized continuous variables further enhanced both the discrimination and reclassification capabilities of the candidate models. Notably, the model incorporating TyG-WWI (Model 3b) demonstrated the most substantial improvement, with the AUC rising to 0.802 (ΔAUC=0.104, DeLong P < 0.001), an IDI of 0.150 (95% CI 0.131–0.170), and a continuous NRI of 0.706 (95% CI 0.600–0.810). Models incorporating TyG (Model 3a), TyG-CVAI (Model 3c), and TyG-BRI (Model 3d) also exhibited improved performance, yielding AUCs of 0.738, 0.747, and 0.730, respectively, with significant gains in both IDI and continuous NRI (**Figure 4**; **Table 4**). Bootstrap bias-corrected calibration curves and decision curve analysis were further used to assess model calibration and clinical net benefit, respectively (**Figure 5**). The calibration curves showed good agreement between predicted probabilities and observed risks, while the model incorporating TyG-WWI demonstrated the highest net benefit across a broad range of threshold probabilities.

To mitigate optimism bias inherent in apparent (in-sample) performance, we further evaluated the models’ discrimination, incremental utility, and clinical net benefit using out-of-fold (OOF) predicted probabilities derived from repeated stratified 10-fold cross-validation (**Supplementary Figures 5**, **6**; **Supplementary Table 2**). Within the OOF framework, the baseline model (Model 1) achieved an AUC = 0.672. The addition of raw-scale AHI (Model 2) increased the AUC to 0.687 (ΔAUC=0.015, DeLong P = 0.037), with an IDI of 0.017 (95% CI 0.010–0.025) and a continuous NRI of 0.236 (95% CI 0.116–0.355). The further inclusion of Z-standardized TyG-related indices consistently improved model performance, where TyG-WWI again provided the largest incremental benefit (AUC = 0.794, ΔAUC=0.108, DeLong P < 0.001; IDI = 0.150, 95% CI 0.129–0.172; continuous NRI = 0.695, 95% CI 0.582–0.806). Consistent incremental gains were also observed for TyG, TyG-CVAI, and TyG-BRI (**Supplementary Table 2**). Furthermore, OOF calibration curves closely aligned with the ideal 45° reference line, indicating excellent agreement between predicted probabilities and observed risks (**Supplementary Figure 6A**). Decision curve analysis (DCA) demonstrated that the model incorporating TyG-WWI achieved higher net benefits across a wide range of clinically relevant threshold probabilities, suggesting its superior potential for risk stratification in the high-risk hospitalized OSAHS population represented in this study (**Supplementary Figure 6B**).

### 3.6 MACE incidence and Kaplan–Meier analysis during follow-up

Among the 1,122 patients followed, 80 major adverse cardiovascular events (MACE) occurred, representing an overall incidence of 7.1%. The composition of MACE was primarily characterized by non-fatal myocardial infarction (2.0%) and stroke (1.5%), as well as cardiac death, hospitalization for heart failure, and malignant arrhythmia (**Supplementary Table 3**).

**Figure 6:**
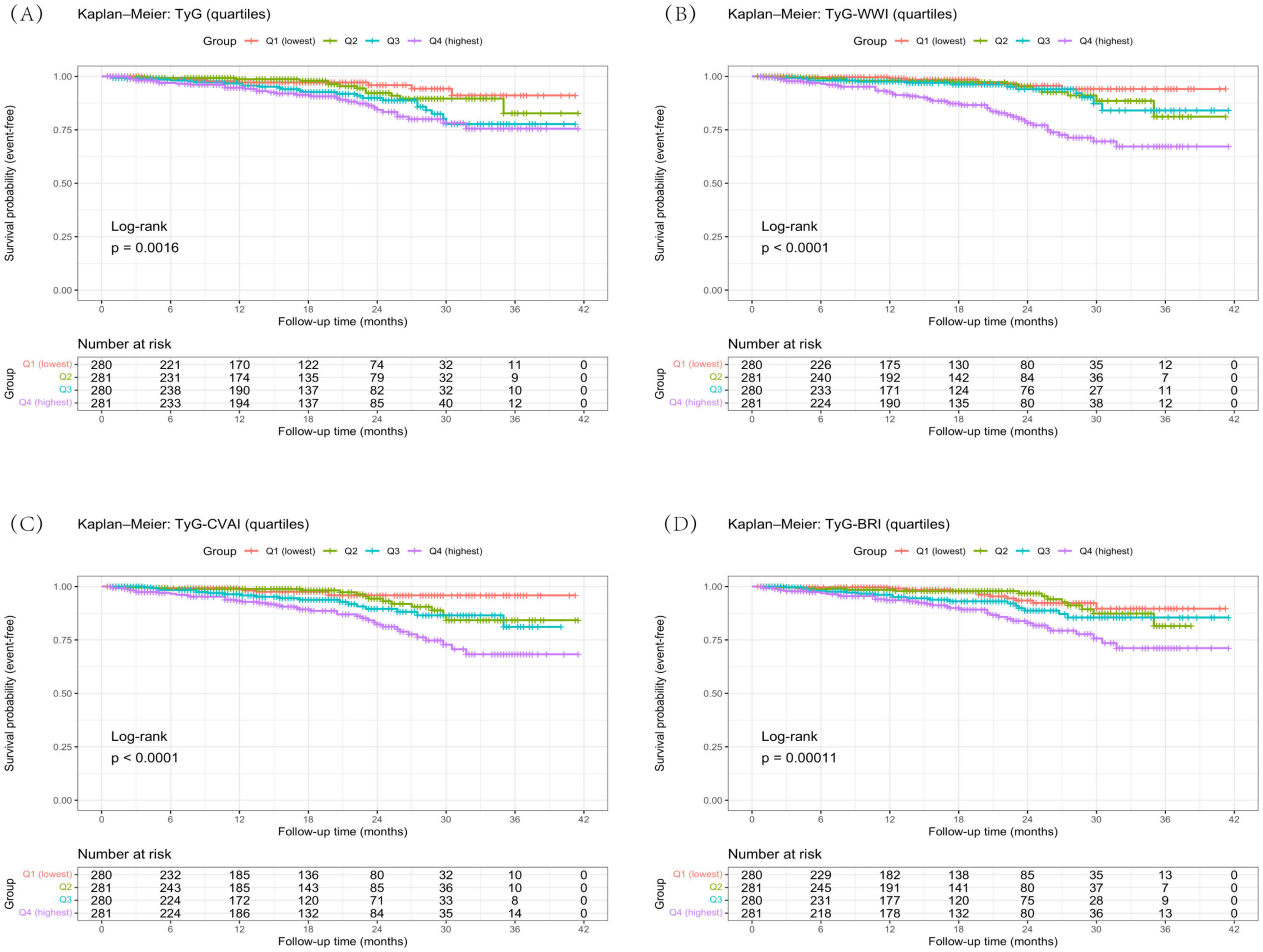
Kaplan–Meier curves illustrate the cumulative incidence of MACE across quartiles (Q1–Q4) of **(A)** TyG, **(B)** TyG-WWI, **(C)** TyG-CVAI, and **(D)** TyG-BRI. Differences between survival curves were evaluated using the log-rank test. A greater degree of curve separation indicates a more robust capacity for MACE risk stratification by the respective index.

Kaplan–Meier (KM) curves were plotted by stratifying the baseline population into quartiles of Z-standardized TyG-WWI (**Figure 6B**). The results demonstrated a progressive increase in the cumulative incidence of MACE as TyG-WWI levels rose. The log-rank test confirmed significant differences between the quartile groups (P < 0.001). Similarly, KM curves grouped by the TyG index alone (**Figure 6A**) also indicated a poorer prognosis for the high-TyG group; however, the separation between the curves was less pronounced than that observed in the TyG-WWI stratification. This suggests that the composite index may offer superior discrimination of high-risk individuals compared to the standalone marker. Consistent trends were observed in the KM analyses for TyG-CVAI and TyG-BRI (**Figures 6C, D**), where the cumulative risk of events was markedly higher in the high-index groups than in the low-index groups.

The KM analysis provides visual evidence of the prognostic discriminative capacity of TyG-related indices. Given that KM curves do not account for confounding factors, adjusted risk estimates are provided in the subsequent Cox regression analysis.

### 3.7 Cox proportional hazards regression analysis of baseline TyG-related indices for MACE

In the unadjusted Cox models (**Table 5**), baseline Z-standardized TyG and its body fat distribution composites (TyG-CVAI, TyG-WWI, and TyG-BRI) were all significantly associated with an increased risk of incident MACE when modeled per 1-standard deviation (1−SD) increase. Among these, TyG-WWI exhibited the most robust association. After adjusting for traditional cardiovascular risk factors (age, sex, hypertension, diabetes, systolic blood pressure, smoking, LDL-C, and white blood cell count), these associations remained statistically significant. However, upon further adjustment for raw-scale AHI in Model 3, the associations for TyG and TyG-BRI were attenuated, whereas TyG-WWI and TyG-CVAI remained independently associated with MACE (TyG-WWI: HR 1.545, 95% CI 1.217–1.961; TyG-CVAI: HR 1.285, 95% CI 1.018–1.622). Nevertheless, because only 80 MACE occurred, these estimates may be vulnerable to overfitting and should be interpreted as supportive rather than definitive prognostic evidence.

**Table 5:** Cox proportional hazards regression analysis of TyG-related indices and MACE risk.

Primary exposure (per 1-SD)	Model 1 HR (95% CI)	P value	Model 2 HR(95% CI)	P value	Model 3 HR (95% CI)	P value
TyG	1.755 (1.397–2.203)	<0.001	1.352 (1.053–1.737)	0.018	1.251 (0.972–1.612)	0.082
TyG-CVAI	1.593 (1.332–1.904)	<0.001	1.417 (1.135–1.768)	0.002	1.285 (1.018–1.622)	0.035
TyG-WWI	1.949 (1.606–2.366)	<0.001	1.665 (1.322–2.097)	<0.001	1.545 (1.217–1.961)	<0.001
TyG-BRI	1.410 (1.195–1.664)	<0.001	1.272 (1.046–1.545)	0.016	1.186 (0.968–1.455)	0.100

**Table 5**. Cox proportional hazards models were used to evaluate the association between a 1-SD increase in Z-standardized TyG, TyG-CVAI, TyG-WWI, and TyG-BRI with MACE risk. Model 1: Unadjusted. Model 2: Adjusted for age, sex, hypertension, diabetes, systolic blood pressure, smoking, LDL-C, and WBC count. Model 3: Adjusted for variables in Model 2 plus raw-scale AHI. The proportional hazards assumption was verified using Schoenfeld residuals.

Given that the expanded MACE composite endpoint includes heterogeneous outcomes such as arrhythmia and heart failure hospitalization, we performed a sensitivity analysis using “Hard MACE” (cardiac death, non-fatal myocardial infarction, stroke, and unplanned revascularization) as the endpoint (**Supplementary Table 4**). In the fully adjusted model (Model 3), TyG-WWI maintained a strong association with Hard MACE (HR = 1.713, 95% CI 1.303–2.251, P < 0.001). Significant associations were also observed for TyG-CVAI (HR = 1.328, 95% CI 1.015–1.737, P = 0.038) and the TyG index (HR = 1.444, 95% CI 1.080–1.930, P = 0.013). Conversely, TyG-BRI did not reach statistical significance in Model 3 (HR = 1.185, 95% CI 0.936–1.499, P = 0.159). Overall, the results for Hard MACE were consistent with the primary findings, suggesting that the observed associations were not driven solely by “soft” endpoints, thereby mitigating concerns regarding the heterogeneity of the composite endpoint.

Quartile analysis in the fully adjusted model (**Supplementary Table 5**) revealed that the TyG index did not exhibit a significant monotonic gradient (P_for trend_ = 0.206). In contrast, subjects in the highest quartiles (Q4) of TyG-CVAI and TyG-WWI had a significantly higher risk of MACE compared to those in the lowest quartiles (Q1) (TyG-CVAI: Q4 vs. Q1, HR 2.798, 95% CI 1.187–6.592, P_for trend_ = 0.006; TyG-WWI: Q4 vs. Q1, HR 3.399, 95% CI 1.473–7.846, P_for trend_ = 0.001). For TyG-BRI, the Q4 vs. Q1 comparison was also significant (HR 2.176, 95% CI 1.038–4.561) with an overall dose-response trend (P_for trend_ = 0.016), suggesting that the increased risk is primarily driven by the upper tail of the distribution. The Schoenfeld residual test showed no evidence of violating the proportional hazards assumption (global P = 0.646; all individual covariates P>0.05).

To address the potential for overfitting due to a relatively low Events Per Variable (EPV) ratio (80 events for 10 parameters; EPV = 8.0; **Supplementary Table 6**), we conducted ridge-penalized Cox regression for the four indices (**Supplementary Table 7**). The penalty parameter λ_min_ was determined via event-stratified 5-fold cross-validation. As the penalty intensity increased, the coefficients exhibited expected shrinkage toward zero (HR → 1). Within the pre-specified range (λ=0–10), the HR directions for the four exposure indices remained consistent: TyG (1.004–1.250), TyG-CVAI (1.004–1.281), TyG-WWI (1.005–1.536), and TyG-BRI (1.003–1.184). Notably, TyG-WWI demonstrated the most stable and highest magnitude of association across varying penalty intensities, reinforcing the robustness of our primary conclusions under regularized estimation.

In summary, after controlling for traditional risk factors, all TyG-related composite indices were associated with an increased risk of MACE. After further adjusting for OSAHS severity (AHI), TyG-WWI and TyG-CVAI continued to provide independent prognostic information, whereas the associations for the standalone TyG index and TyG-BRI were more susceptible to attenuation by AHI.

### 3.8 Time-dependent ROC analysis and temporal discrimination

**Figure 7:**
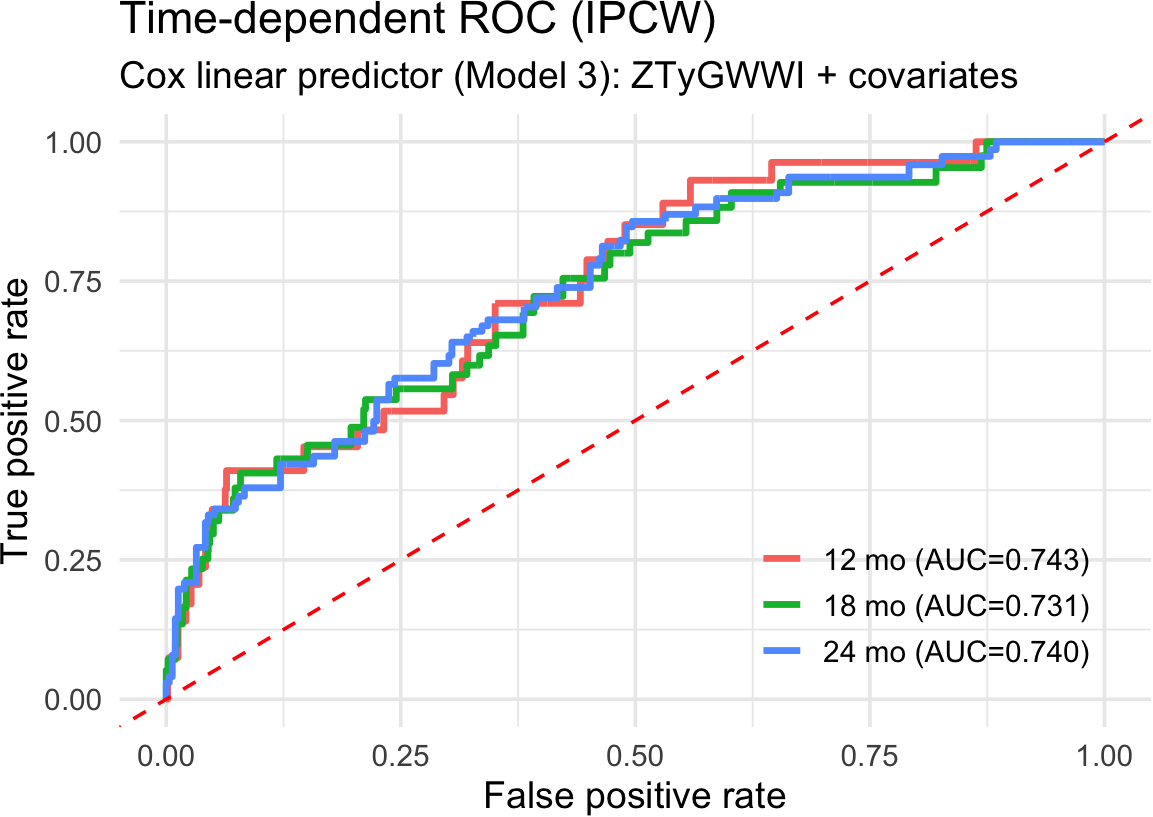
Time-dependent ROC curves were constructed based on the linear predictors of the Cox models using the inverse probability of censoring weighting (IPCW) method to evaluate the dynamic discriminative capacity for MACE at predefined follow-up intervals (12, 18, and 24 months). The AUCs of each candidate model at these time points are presented to illustrate the temporal stability of their predictive performance.

To evaluate the temporal evolution of time-dependent discrimination, we compared the time-dependent ROC curves derived from the linear predictors of the Cox models using inverse probability of censoring weighting (IPCW) (**Figure 7**, **Supplementary Figure 7**). The results showed that at the 12-, 18-, and 24-month time points, the model incorporating Z-standardized TyG-WWI consistently achieved the highest AUC values (0.743, 0.731, and 0.740, respectively). These findings suggest that the TyG-WWI model showed relatively stable discriminative performance throughout the follow-up period.

In comparison, the AUC values for the model incorporating Z-standardized TyG-CVAI were 0.712, 0.705, and 0.718 at the same intervals, remaining slightly lower than those of the TyG-WWI model. Overall, the model integrated with Z-standardized TyG-WWI exhibited higher and relatively stable time-dependent discrimination across follow-up stages, suggesting internally supportive temporal discrimination for MACE risk stratification.

### 3.9 Exploratory decomposition of the AHI–MACE association through TyG-related indices

**Figure 8:**
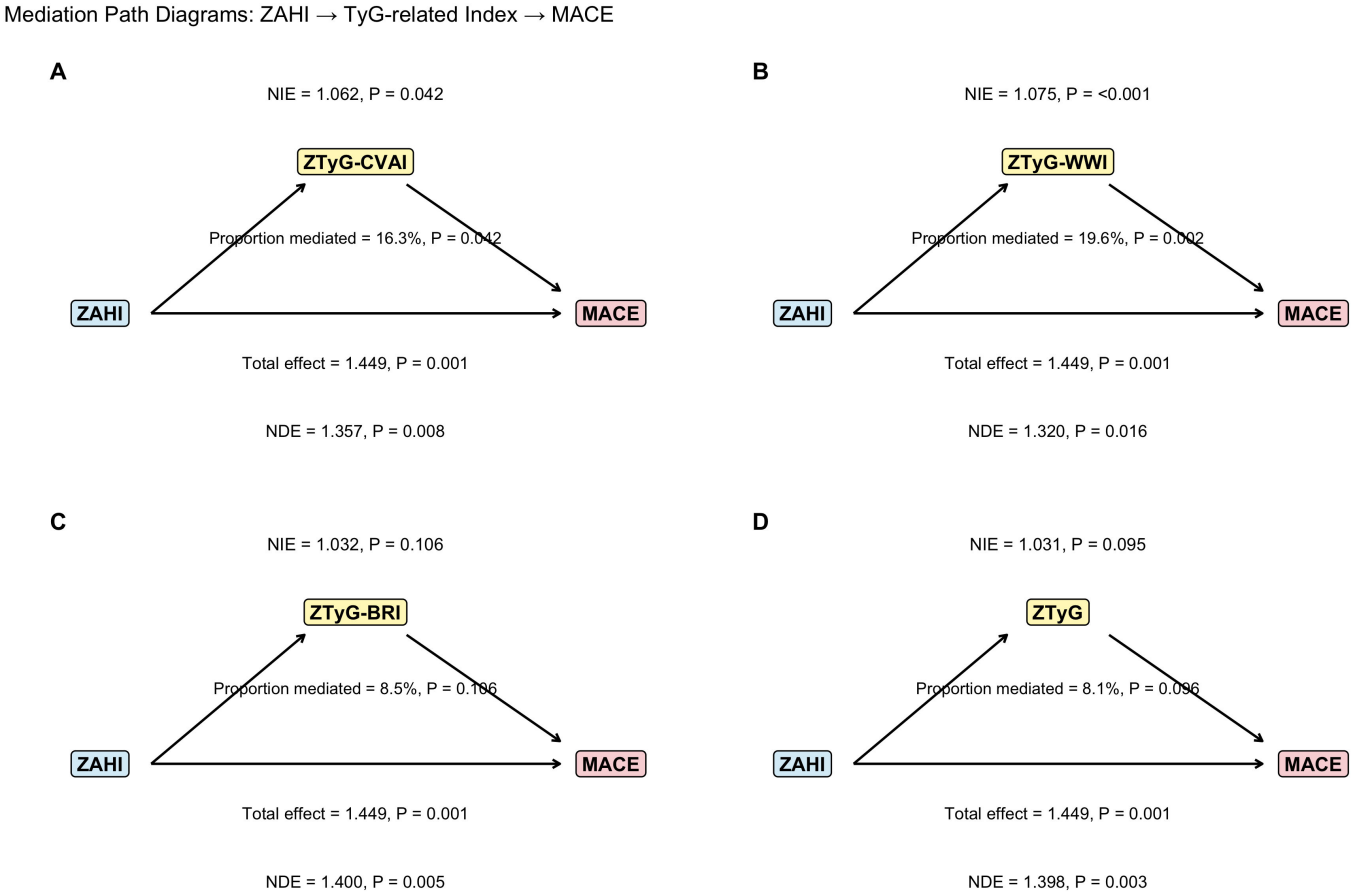
Exploratory statistical decomposition of the association between Z-standardized AHI (ZAHI) and incident MACE. **(A)** ZTyG-CVAI, **(B)** ZTyG-WWI, **(C)** ZTyG-BRI, and **(D)** ZTyG were evaluated separately as candidate pathway variables to estimate the total effect (TE), natural direct effect (NDE), and natural indirect effect (NIE). The figure presents effect estimates for each candidate pathway variable to describe potential indirect pathway signals. Because ZAHI and TyG-related candidate pathway variables were measured concurrently at baseline, this analysis should be interpreted as exploratory statistical decomposition rather than definitive causal mediation evidence.

Under a counterfactual-based exploratory statistical decomposition framework, we examined the association between Z-standardized AHI (ZAHI) and incident MACE during follow-up. Z-standardized TyG, TyG-WWI, TyG-CVAI, and TyG-BRI were evaluated separately as candidate pathway variables, and the total effect (TE), natural direct effect (NDE), and natural indirect effect (NIE) were estimated (**Figure 8**). The overall association between ZAHI and MACE risk was statistically significant in the decomposition models (TE = 1.449, P = 0.001). When TyG-WWI was specified as the candidate pathway variable, the strongest indirect pathway signal was observed, with an NIE of 1.075 (P < 0.001) and an estimated proportion explained of 19.6% (P = 0.002); the NDE also remained statistically significant (1.320, P = 0.016), indicating a pattern consistent with partial statistical decomposition rather than confirmed mediation. When TyG-CVAI was evaluated, a weaker but statistically significant indirect pathway signal was observed (NIE = 1.062, P = 0.042), with an estimated proportion explained of 16.3% (P = 0.042), while the NDE remained significant (1.357, P = 0.008). By contrast, the NIE estimates for TyG and TyG-BRI did not reach statistical significance (TyG: NIE = 1.031, P = 0.095; TyG-BRI: NIE = 1.032, P = 0.106), suggesting limited evidence for indirect pathway signals involving these two indices in the present exploratory analysis.

Given that AHI and oxygenation metrics, as well as the candidate pathway variables, were measured concurrently at baseline, and that observational data cannot verify key causal identification assumptions, including the absence of unmeasured confounding, these results should be interpreted as exploratory statistical decomposition signals. They should not be construed as verified causal mediation effects or definitive mediation proportions.

To assess robustness to model specification and covariate handling, we further conducted prespecified sensitivity analyses, including additional adjustment for ALT, replacement of LDL-C with TC, and repeated modeling using Z-standardized LSaO2 or MSaO2 in place of ZAHI because of the substantial correlation among sleep severity indicators (**Supplementary Figure 8**). Overall, the sensitivity analyses were directionally consistent with the primary analysis: the TyG-WWI-related signal was the most stable, with the estimated proportion explained remaining approximately 16.8%–19.1% across model specifications; TyG-CVAI ranked second, with estimates ranging from approximately 12.6% to 16.8% and remaining statistically significant in most analyses. By contrast, the signals for TyG and TyG-BRI were weak and generally non-significant. These findings suggest that TyG-WWI, and to a lesser extent TyG-CVAI, may capture more consistent statistical pathway information linking OSAHS severity to residual cardiovascular risk; however, this interpretation requires validation in studies with repeated longitudinal measurements and stronger causal identification designs.

## 4 Discussion

This study systematically compared the associations of the triglyceride-glucose (TyG) index and its body fat distribution composites (TyG-WWI, TyG-CVAI, and TyG-BRI) with prevalent coronary heart disease (CHD)and incident major adverse cardiovascular events (MACE) within a cohort of adult inpatients with obstructive sleep apnea-hypopnea syndrome (OSAHS) who underwent concurrent polysomnography (PSG) and coronary angiography (CAG). We further evaluated their incremental discriminative value beyond traditional cardiovascular risk factors and the apnea-hypopnea index (AHI), and performed an exploratory counterfactual-based statistical decomposition to assess potential indirect pathway signals in the AHI–MACE association. Our findings suggest that TyG-related indices, particularly TyG-WWI, were consistently associated with CHD prevalence and provided incremental discrimination and reclassification information. TyG-WWI also showed supportive longitudinal associations with incident MACE, although these prognostic findings should be interpreted cautiously because of the limited number of events. Exploratory decomposition further suggested indirect pathway signals involving TyG-WWI and TyG-CVAI, but these findings should be regarded as hypothesis-generating rather than causal mediation evidence.

### 4.1 Robustness and phenotypic interpretation

To rigorously test the robustness and reproducibility of these findings, we performed multi-dimensional consistency assessments across various analytical frameworks. TyG-WWI maintained stable effect directions and magnitudes in cross-sectional CHD logistic regression models, longitudinal Cox regression, and sensitivity analyses using “Hard MACE” endpoints (cardiac death, non-fatal myocardial infarction, stroke, or revascularization). Concurrently, it provided the most substantial incremental information in terms of model discriminative performance. This consistency suggests that the superior performance of TyG-WWI is not an artifact of any single analytical approach but represents a reproducible risk stratification signal.

From a phenotypic perspective, the TyG index primarily reflects the metabolic burden related to insulin resistance, while the weight-adjusted waist index (WWI) supplements this with information on fat distribution and central obesity. Consequently, TyG-WWI integrates cumulative metabolic and morphological risk phenotypes more comprehensively than single-dimension indices, better reflecting the composite exposure intensity associated with cardiovascular outcomes in an OSAHS population enriched with obesity and metabolic derangements. This interpretation remains at the phenotypic level and necessitates independent validation in external cohorts.

### 4.2 Non-linear associations and threshold effects

In the cross-sectional analysis, the association strength of TyG-WWI with CHD was significantly higher than that of the standalone TyG index or other composites. After full adjustment and Z-standardization, the odds ratio (OR) per 1-standard deviation (1-SD) increase reached 3.21 (95% CI 2.68–3.89), with quartile analysis revealing a clear dose-response gradient (Q4 vs. Q1 OR = 18.84,95% CI 11.84–30.72). Restricted cubic splines (RCS) and two-piecewise logistic regression further revealed significant non-linear relationships for TyG, TyG-WWI, and TyG-BRI (with inflection points at Z=−1.089,−0.407, and 0.241, respectively), where the piecewise models outperformed linear models in goodness-of-fit.

These threshold-like patterns suggest that cardiometabolic risk accelerates significantly once the combined metabolic-adiposity burden exceeds a compensatory threshold. Although the sparsity of events at the lower tail of the TyG distribution resulted in less stable estimates, the overall non-linear signals remained robust across sensitivity tests. This highlights that the risk effect of TyG-related indices is not uniform across their distribution—an insight crucial for future risk-stratification algorithms.

### 4.3 Incremental discrimination and clinical utility

Incremental discrimination analysis showed that adding raw-scale AHI to the traditional risk factor model improved the Area Under the Curve (AUC) from 0.683 to 0.698. The subsequent inclusion of Z-standardized TyG composites further enhanced model performance, with TyG-WWI providing the largest gain (AUC = 0.802,ΔAUC=0.104, continuous NRI = 0.706,IDI=0.150; all P<0.001). Internal validation via Bootstrapand repeated stratified 10-fold cross-validation confirmed these increments while mitigating optimism bias.

These results suggest that in the clinical setting of hospitalized OSAHS with high pre-test probability, TyG-WWI provides clinically meaningful risk reclassification information. However, since the index was derived within this specific cohort, TyG-WWI should be viewed as a promising candidate for stratification. Even with internal validation, its clinical deployment requires rigorous external validation rather than immediate application.

### 4.4 Longitudinal associations and temporal discrimination

The longitudinal analysis provided supportive evidence for the association between TyG-WWI and subsequent MACE. During a median follow-up of 17 months, 80 MACE occurred, corresponding to an incidence of 7.1%. Kaplan–Meier curves showed a graded separation across TyG-WWI quartiles. In the fully adjusted Cox model, TyG-WWI remained associated with incident MACE (HR = 1.545, 95% CI 1.217–1.961), whereas the association for the standalone TyG index was attenuated after adjustment for AHI. However, given the limited number of events and the relatively low events-per-variable ratio, these prognostic estimates should be interpreted cautiously. Ridge-penalized Cox regression showed directionally consistent estimates, particularly for TyG-WWI, but this sensitivity analysis cannot fully eliminate the possibility of model overfitting.

Time-dependent ROC analysis using inverse probability of censoring weighting showed that the TyG-WWI model had relatively stable discrimination at 12, 18, and 24 months, with AUCs of 0.743, 0.731, and 0.740, respectively. Although these values were higher than those of the other evaluated indices, the results should be regarded as internally supportive rather than externally validated evidence. Similarly, the Hard MACE sensitivity analysis yielded generally consistent results, suggesting that the observed associations were not solely driven by softer endpoint components. Nevertheless, larger prospective cohorts with longer follow-up and external validation are required before TyG-WWI can be considered a reliable prognostic tool for MACE risk stratification.

Under the counterfactual framework, exploratory statistical decomposition using Z-standardized AHI as the exposure and Z-standardized TyG-related composite indices as candidate pathway variables provided hypothesis-generating pathway-level information. The results showed that both TyG-WWI and TyG-CVAI exhibited statistically significant natural indirect effects, while their natural direct effects also remained statistically significant, a decomposition pattern consistent with partial indirect pathway contribution. In contrast, the natural indirect effects of TyG and TyG-BRI did not reach statistical significance, suggesting relatively limited evidence for their indirect pathways. Multiple sensitivity analyses, including additional adjustment for ALT, replacement of LDL-C with TC, and substitution of AHI with mean oxygen saturation or lowest oxygen saturation, yielded directionally consistent findings overall: the indirect pathway signal for TyG-WWI was the most stable, followed by TyG-CVAI, whereas the indirect effects of TyG and TyG-BRI were generally weaker and mostly failed to reach statistical significance.

However, these findings must be interpreted with strict caution. Because the exposure (AHI or oxygenation metrics) and candidate mediators (TyG and its composites) were measured concurrently at baseline, the temporal sequence cannot be empirically confirmed. Furthermore, observational studies struggle to fully satisfy the critical identification assumptions of counterfactual decomposition, such as the absence of unmeasured confounding. Therefore, these results are better understood as statistical decomposition signals and hypothesis-generating evidence consistent with potential mechanisms, rather than definitive causal mediation inferences. Although MACE outcomes were determined prospectively during follow-up, this only supports the direction of a prognostic association between baseline phenotypes and subsequent clinical outcomes, which is insufficient to establish a causal pathway in isolation. Future research utilizing repeated longitudinal measurements and designs with stronger causal identification capabilities is required to further validate the temporal relationships and underlying mechanisms between OSAHS severity, metabolic-adiposity phenotypes, and cardiovascular events.

## 5 Strengths

Compared with previous research, the results of this study are consistent with the overall body of evidence linking the triglyceride-glucose (TyG) index and its related composite indices to elevated cardiovascular risk (21, 22). Furthermore, our findings suggest that within the obstructive sleep apnea-hypopnea syndrome (OSAHS) population—a group characterized by a high prevalence of metabolic derangements and adiposity phenotypes—the composite indices integrating the TyG index with body fat distribution metrics, particularly TyG-WWI, may provide significantly greater incremental gains in risk stratification compared to the TyG index alone (8, 22). While previous epidemiological studies have extensively explored the relationship between TyG or its derivatives and adverse cardiovascular outcomes such as long-term MACE, cardiovascular mortality, and stroke, most evidence stems from the general population, metabolic cohorts, or other high-risk cardiovascular groups, and the relative superiority of specific indices has been inconsistent across studies (21, 22).

Within the OSAHS context, existing research has demonstrated that the TyG index is associated with OSAHS risk, disease severity, and increased cardiovascular risk; in higher-risk clinical scenarios such as OSAHS comorbid with acute coronary syndrome (ACS), a synergistic effect between high TyG levels and OSAHS may further exacerbate adverse cardiovascular outcomes (23, 24). However, most current studies focus on a single TyG marker, isolated composite parameters, or general cardiovascular risk outcomes. Head-to-head evidence systematically comparing multiple TyG-related composite indices for the prediction of CHD prevalence and subsequent MACE risk in OSAHS patients undergoing concurrent polysomnography (PSG) and coronary angiography (CAG) remains limited (4, 24). Consequently, this study performed a parallel comparison of TyG, TyG-WWI, TyG-CVAI, and TyG-BRI within the same cohort, further adjusting for apnea-hypopnea index (AHI) on top of traditional risk factors while conducting rigorous sensitivity analyses and internal validation. The results indicate that TyG-WWI exhibits the most stable performance across cross-sectional CHD identification, longitudinal MACE prediction, and incremental discrimination, providing targeted clinical evidence for refined risk stratification based on metabolic-adiposity phenotypes in the OSAHS population.

Specifically, our findings are supported by observations across various research settings. Prospective studies in cardiovascular-kidney-metabolic (CKM) syndrome or general populations have indicated that elevated TyG-related indices are associated with increased CVD and mortality risk. In OSAHS populations, the TyG index has also been independently linked to higher lifetime cardiovascular risk, suggesting its robust potential for risk stratification within the context of sleep-disordered breathing (4, 25). Consistent with the findings of Qiu et al., who observed independent associations between the TyG index and both CHD risk and coronary atherosclerosis severity in 1,059 patients with OSA (11), our study demonstrates a stable dose-response relationship between TyG (and its composites) and CHD risk. Furthermore, Zhang et al. confirmed in an OSA-ACS cohort that the coexistence of high TyG levels and OSAHS significantly increases the risk of subsequent adverse cardiovascular events (13), supporting our longitudinal MACE risk findings from a different clinical perspective. Li et al. reported an independent association between the TyG index and OSAHS severity in hypertensive patients (26), suggesting a tight link between sleep-breathing burden and metabolic-adiposity phenotypes. Moreover, Yue et al. demonstrated in an early-stage CKM population that novel composite markers like TyG-CVAI, TyG-WWI, and TyG-BRI offer richer prognostic information than the TyG index alone (8), which aligns with the overall trend observed in our study.

In addition to TyG-based indices, the triglyceride-to-high-density lipoprotein cholesterol ratio (TG/HDL-C) has also been proposed as a readily available lipid-derived marker of insulin resistance and atherogenic dyslipidemia. Di Marco et al. recently reported that TG/HDL-C was independently associated with arterial stiffness assessed by pulse wave velocity in individuals with prediabetes, whereas TyG was not independently associated with this vascular phenotype. These findings suggest that TG/HDL-C and TyG-related indices may capture partially overlapping but not identical metabolic-risk domains, and future studies should directly compare their incremental value for cardiovascular risk stratification in OSAHS (27).

Similarly, evidence from familial hypercholesterolemia (FH), a population with genetically driven lifelong LDL-C exposure, further supports the relevance of metabolic and insulin-resistance-related phenotypes in atherosclerotic risk. Bosco et al. reported that FH subjects with subclinical atherosclerosis had higher TG/HDL-C and TyG values than those without subclinical atherosclerosis, together with a more impaired innate immune profile. These findings suggest that IR-related metabolic abnormalities may act as risk modifiers beyond traditional lipid burden and may contribute to endothelial injury and atherosclerotic progression. Although FH differs pathophysiologically from OSAHS, this evidence reinforces the broader concept that lipid-glucose metabolic dysfunction is closely linked to vascular injury across high-risk cardiovascular populations (28).

To our knowledge, this is among the first studies to systematically compare TyG, TyG-WWI, TyG-CVAI, and TyG-BRI in an OSAHS cohort evaluated by both PSG and CAG. By incorporating internal validation, time-dependent ROC analysis, sensitivity analysis for “Hard MACE,” and exploratory statistical decomposition, TyG-WWI showed the most consistent performance across CHD and MACE-related analyses. While prior studies suggested that metabolic composites might partially mediate the relationship between OSA and downstream outcomes like hypertension or fatty liver (29, 30), our study extends this mediation logic to the AHI-MACE axis, quantifying the partial indirect effect of TyG-WWI and providing new insights into the metabolic-adiposity pathways underlying residual cardiovascular risk in OSAHS.

From a clinical perspective, TyG-WWI may serve as a simple and cost-effective candidate marker to support risk stratification in selected high-risk hospitalized OSAHS patients undergoing coronary evaluation. However, its applicability to broader OSAHS populations requires validation in prospective multicenter cohorts. Rather than supporting immediate clinical implementation, our findings suggest that TyG-WWI may help identify patients who could benefit from closer cardiometabolic assessment and integrated management of metabolic risk factors and OSAHS. Pathophysiologically, these findings support future hypothesis-driven studies to investigate whether longitudinal changes in this composite phenotype are associated with residual MACE risk. Future research should evaluate the trajectories of TyG-WWI, integrate multimodal adiposity imaging, and determine whether targeted metabolic-adiposity management improves hard cardiovascular outcomes.

## 6 Limitations and weaknesses

First, this was a retrospective, single-center observational study conducted in hospitalized OSAHS patients who completed both clinically indicated coronary angiography (CAG) and polysomnography (PSG). Because inclusion was non-random and likely driven by clinical suspicion of CHD, non-invasive test results, institutional workflows, and referral pathways, the cohort represented a selected high-risk population rather than a general OSAHS sample. These factors may be associated with both metabolic-adiposity phenotypes, such as TyG-WWI, and CHD or subsequent cardiovascular events; therefore, referral bias, indication bias, and potential collider bias cannot be excluded. Because detailed referral-pathway variables, CAG indications, and pre-test probability estimates were unavailable, these biases could not be fully adjusted for. Accordingly, our conclusions should be restricted to selected high-risk hospitalized OSAHS patients undergoing coronary evaluation, and generalization to community-based OSAHS populations should be made with caution. External validation in prospective multicenter and more representative cohorts is warranted.

Second, due to data limitations, we were unable to systematically quantify and include CPAP therapy adherence, the intensity of lipid-lowering, glucose-lowering, or antihypertensive treatments, or lifestyle factors in the models. Thus, residual confounding is inevitable, and the results should be interpreted as associations between phenotypic markers and CHD/cardiovascular events rather than inferences of treatment effects.

Third, TG/HDL-C was not included in the prespecified comparative framework. TG/HDL-C is a simple surrogate marker of insulin resistance and atherogenic dyslipidemia, and recent evidence suggests its potential value for vascular stiffness and cardiovascular risk assessment. Future studies should directly compare TG/HDL-C with TyG-WWI and other TyG-related adiposity composites to determine whether it provides additional discrimination, reclassification, or clinical utility in OSAHS populations.

Fourth, although bootstrap calibration and penalized regression were used, the longitudinal MACE analysis was based on only 80 events, which may limit model stability and increase the risk of overfitting. In addition, external validation was not performed. Therefore, the MACE-related findings should be interpreted as supportive prognostic evidence rather than definitive prediction evidence, and TyG-WWI should currently be regarded as a candidate stratification marker.

Fifth, the mediation analysis should be interpreted strictly as exploratory and hypothesis-generating. Because AHI and TyG-related candidate mediators were measured concurrently at baseline, their temporal sequence could not be established. Moreover, the observational design cannot fully verify the assumptions required for causal mediation analysis, including the absence of unmeasured exposure–mediator, mediator–outcome, and exposure–outcome confounding. Therefore, the estimated indirect effects should be regarded as exploratory statistical decomposition signals rather than definitive causal mediation evidence. Future studies with repeated longitudinal measurements or interventional designs are needed to validate these potential pathways.

## 7 Conclusion

In summary, in this selected high-risk cohort of hospitalized adults with OSAHS undergoing coronary evaluation, TyG-WWI showed the most consistent association with prevalent CHD among the evaluated TyG-related indices and provided incremental discrimination and reclassification information beyond traditional risk factors and AHI. TyG-WWI also showed supportive longitudinal associations with follow-up MACE; however, these prognostic findings should be interpreted cautiously because the MACE analysis was based on a limited number of events and requires validation in larger prospective multicenter cohorts. Exploratory statistical decomposition suggested potential indirect pathway signals involving TyG-WWI and TyG-CVAI in the AHI–MACE association, but these findings should be regarded as hypothesis-generating rather than causal evidence. Future studies should incorporate external validation, repeated measurements of metabolic-adiposity phenotypes, treatment adherence data, and more representative OSAHS populations to clarify the clinical relevance and outcome implications of these markers.

## Data Availability

The raw data supporting the conclusions of this article will be made available by the authors, without undue reservation.
