# Supplementary material for: Association of the TyG index and its body fat distribution composites with CHD and MACE risk in adults with OSAHS: incremental discrimination and exploratory mediation analysis
Source: Front Endocrinol (Lausanne). 2026 Jun 16;17:1848218. doi: 10.3389/fendo.2026.1848218 (PMC13314442; doi:10.3389/fendo.2026.1848218)
Supplement: Supplementary file 1 [file DataSheet1.docx]

Figure S1: Spearman correlation matrix of TyG-related indices


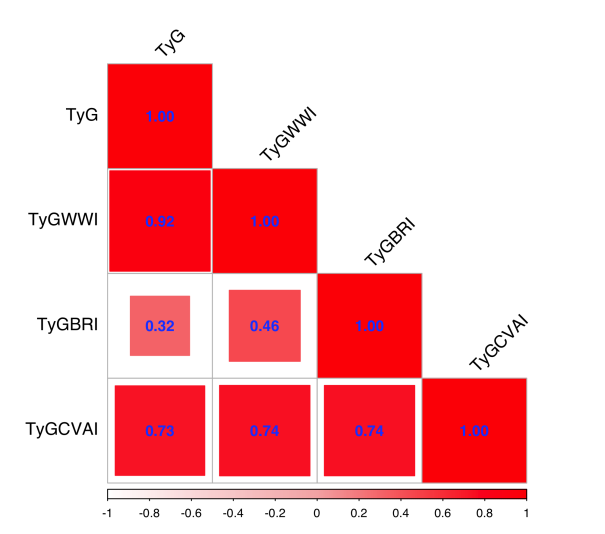


**Figure S1.** Heatmap illustrating the Spearman correlation coefficients between the TyG index and its composite indices (TyG-WWI, TyG-CVAI, and TyG-BRI). Color intensity represents the strength of the correlation, while the color hue indicates the direction (positive or negative). The numerical values within the plot denote the corresponding correlation coefficients. This figure is employed to evaluate the correlation structure of the variables and to identify potential multicollinearity.

Figure S2: CHD-related feature selection results based on the Boruta algorithm


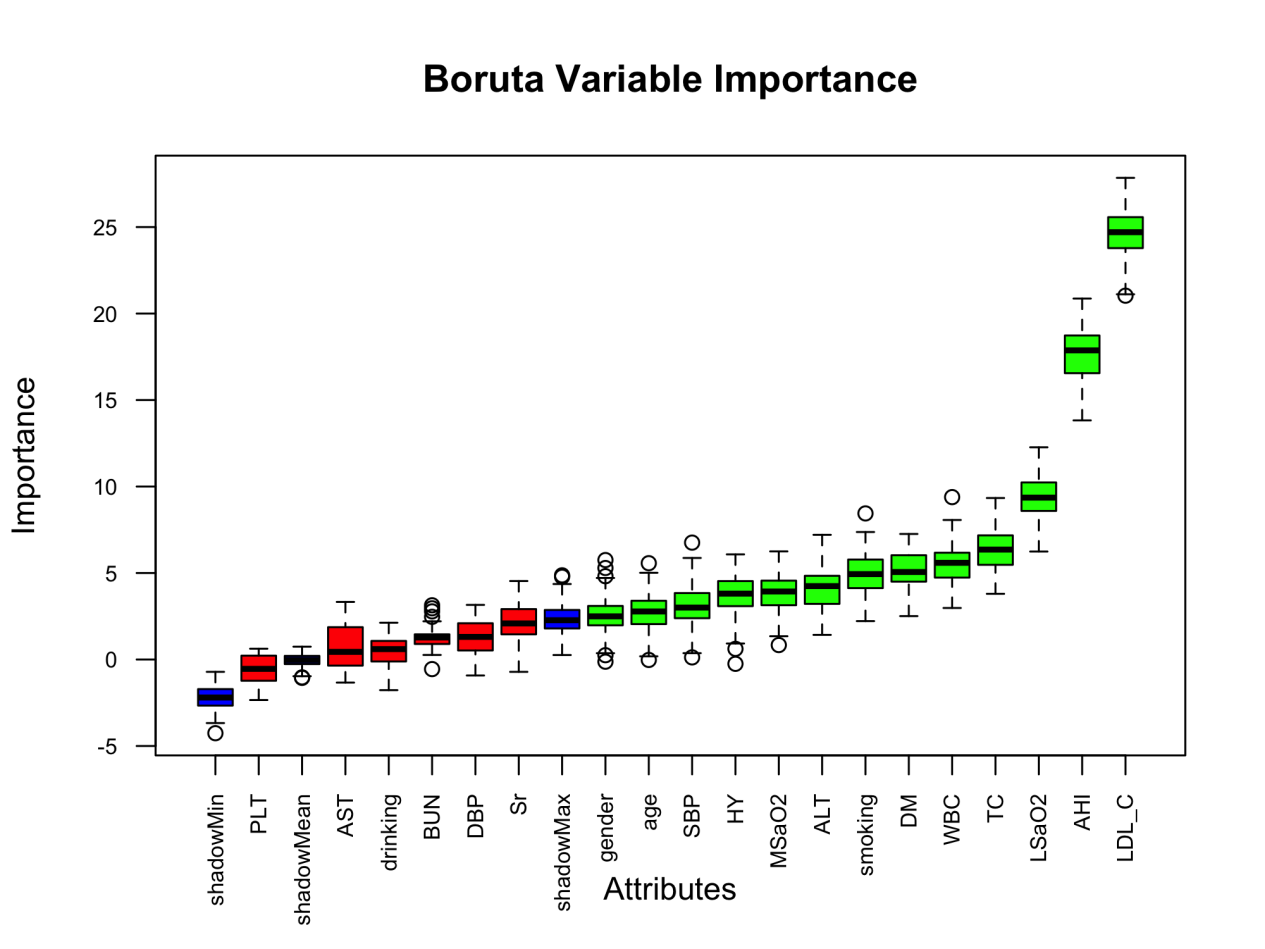


**Figure S2.** The Boruta feature selection algorithm was employed to assess the relative importance of candidate variables for CHD classification. Green indicates confirmed important features, red indicates rejected features, and yellow indicates tentative features. Blue-shaded areas or boxplots represent shadow features generated via random permutation, serving as the reference baseline. This analysis is intended as an exploratory reference and does not replace the pre-specified covariate adjustment strategy used in the primary analysis.

Table S1: Sensitivity analysis of the association between TyG-related indices and CHD

| **Exposure** | **Model specification** | **OR (95% CI)** | **P value** |
| --- | --- | --- | --- |
| **TyG** | Primary analysis (Model 3 + AHI) | 1.84 (1.58–2.15) | <0.001 |
|  | Model 3 + ALT | 1.87 (1.60–2.18) | <0.001 |
|  | Model 3 with TC replacing LDL-C | 1.84 (1.57–2.15) | <0.001 |
|  | Model 2 + MSaO₂ instead of AHI | 1.90 (1.63–2.21) | <0.001 |
|  | Model 2 + LSaO₂ instead of AHI | 1.89 (1.62–2.19) | <0.001 |
| **TyG-CVAI** | Primary analysis (Model 3 + AHI) | 2.07 (1.75–2.44) | <0.001 |
|  | Model 3 + ALT | 2.10 (1.78–2.48) | <0.001 |
|  | Model 3 with TC replacing LDL-C | 2.06 (1.75–2.42) | <0.001 |
|  | Model 2 + MSaO₂ instead of AHI | 2.18 (1.85–2.56) | <0.001 |
|  | Model 2 + LSaO₂ instead of AHI | 2.14 (1.82–2.52) | <0.001 |
| **TyG-WWI** | Primary analysis (Model 3 + AHI) | 3.21 (2.67–3.87) | <0.001 |
|  | Model 3 + ALT | 3.30 (2.73–3.98) | <0.001 |
|  | Model 3 with TC replacing LDL-C | 3.22 (2.67–3.88) | <0.001 |
|  | Model 2 + MSaO₂ instead of AHI | 3.31 (2.75–3.98) | <0.001 |
|  | Model 2 + LSaO₂ instead of AHI | 3.26 (2.71–3.93) | <0.001 |
| **TyG-BRI** | Primary analysis (Model 3 + AHI) | 1.71 (1.46–2.00) | <0.001 |
|  | Model 3 + ALT | 1.72 (1.47–2.02) | <0.001 |
|  | Model 3 with TC replacing LDL-C | 1.71 (1.46–2.00) | <0.001 |
|  | Model 2 + MSaO₂ instead of AHI | 1.78 (1.53–2.09) | <0.001 |
|  | Model 2 + LSaO₂ instead of AHI | 1.76 (1.50–2.06) | <0.001 |

**Table S1.** Multivariate logistic regression was used to evaluate the robustness of the association between CHD and a 1-SD increase in Z-score standardized TyG, TyG-CVAI, TyG-WWI, and TyG-BRI. The primary analysis was based on Model 3 (Model 2 + original scale AHI). Additional sensitivity analyses included further adjustment for ALT, replacement of LDL-C with TC, and replacement of AHI with MSaO₂ or LSaO₂. Odds ratios (OR), 95% confidence intervals (CI), and P-values are reported.

Figure S3: Consistency analysis of the association between TyG-related indices and CHD across subgroups

**
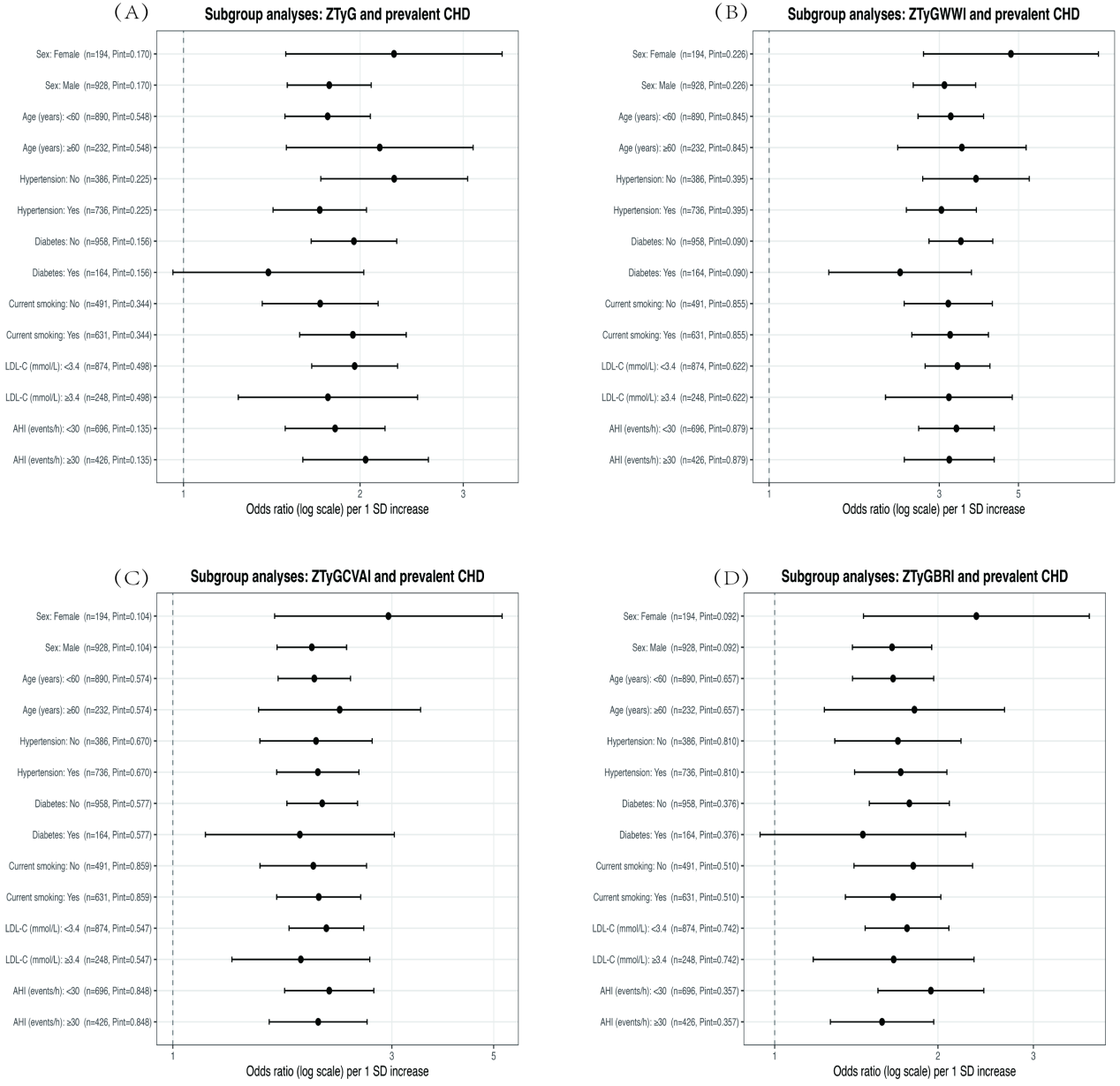
**

**Figure S3.** Forest plots illustrate the effect estimates for the association between TyG-related indices and CHD across various subgroups, including sex, age (<60 or ≥60 years), hypertension, diabetes, smoking status, LDL-C levels (<3.4 or ≥3.4 mmol/L), and OSAHS severity (AHI <30 or ≥30 events/h). Points represent effect estimates, horizontal lines represent 95% CIs, and the dashed vertical line indicates the null effect. Where interaction test results are provided, P for interaction is also labeled.

Figure S4: Incidence of coronary heart disease (CHD) by quartiles (Q1–Q4) of TyG-related indices


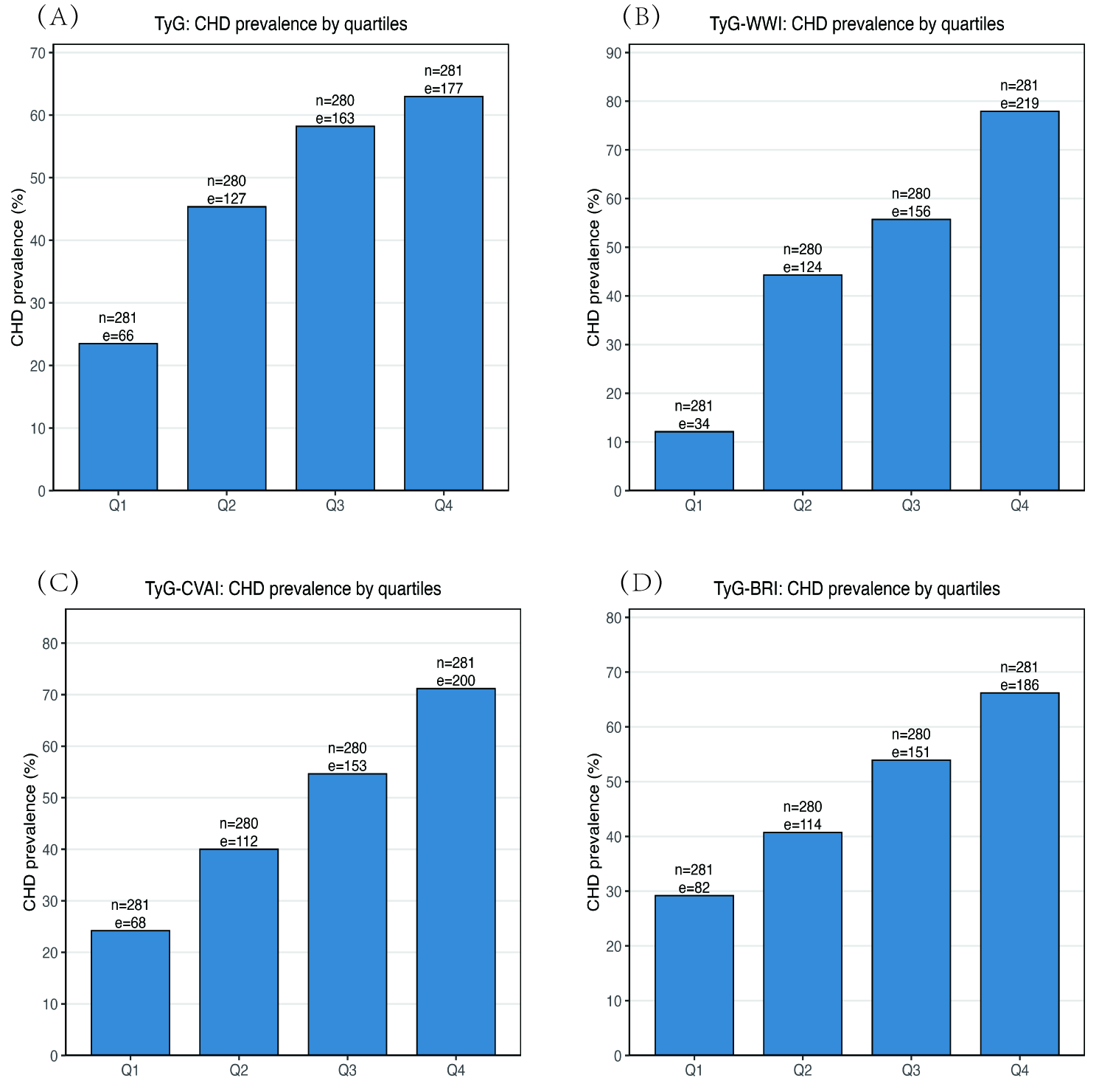


**Figure S4.** CHD incidence rates categorized by quartiles (Q1–Q4) of TyG-related indices. A, TyG; B, TyG-WWI; C, TyG-CVAI; D, TyG-BRI. Bar charts represent the intra-group CHD incidence (%), with sample sizes (n) and the number of CHD events labeled above the bars. Quartile groupings are based on the distribution of each index within the study sample.

Figure S5: ROC curves based on out-of-fold predictions from repeated stratified 10-fold cross-validation

**
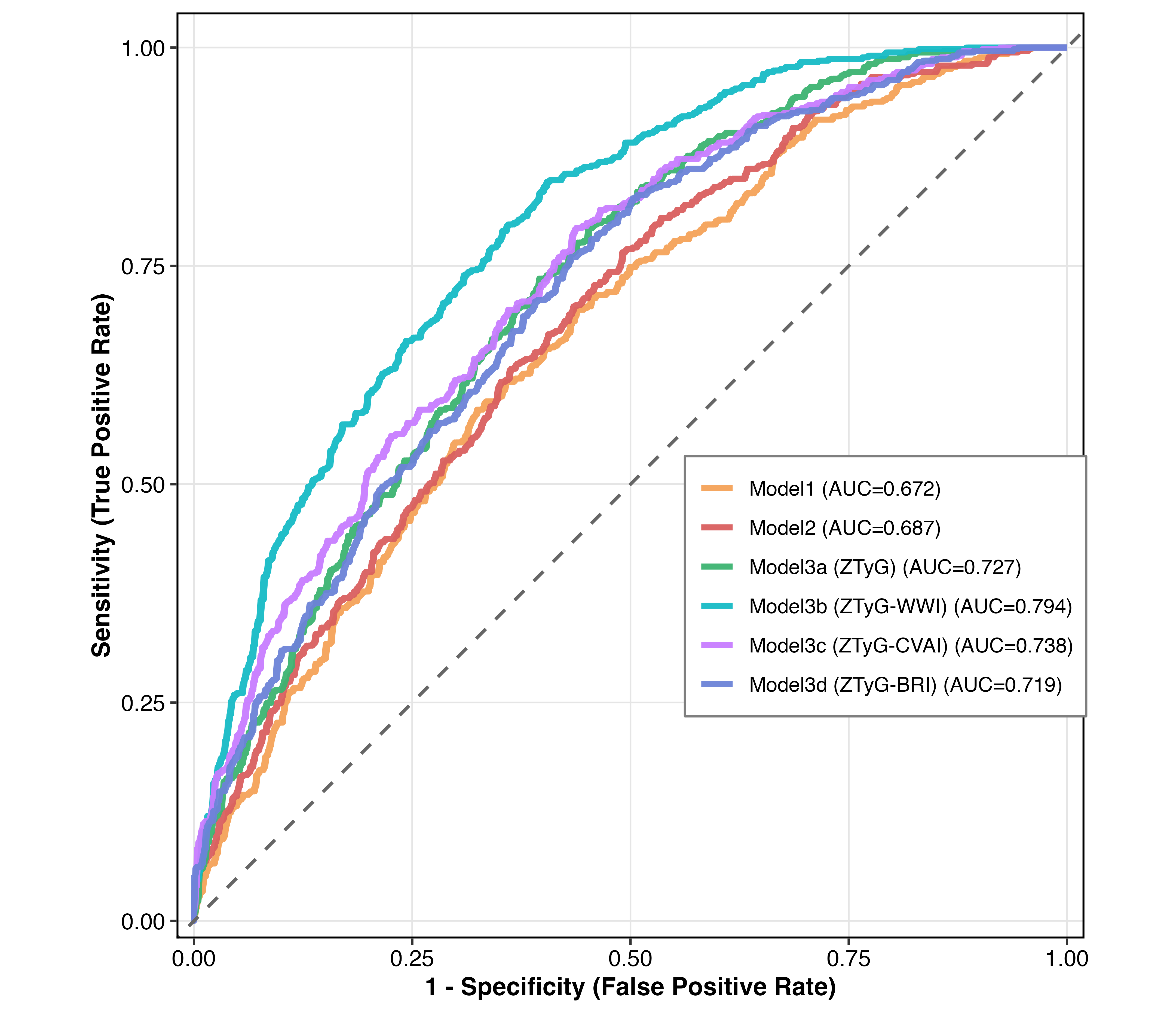
**

**Figure S5.** Receiver Operating Characteristic (ROC) curves were plotted using out-of-fold predictions generated from repeated stratified 10-fold cross-validation to evaluate the discriminatory power of different nested models for CHD. Each curve corresponds to a candidate model, and the Area Under the Curve (AUC) is used to quantify the ability to distinguish CHD from non-CHD individuals. This analysis was performed to minimize optimism bias associated with single-sample internal evaluations.

Table S2: Comparison of discriminatory performance among nested models based on out-of-fold predictions

| **Comparison** | **Reference model AUC** | **New model AUC** | **ΔAUC** | **DeLong p** | **IDI (95%CI)** | **Continuous NRI (95% CI)** |
| --- | --- | --- | --- | --- | --- | --- |
| Model1 → Model 2 | 0.672 | 0.687 | 0.015 | 0.037 | 0.017 (0.010–0.025) | 0.236 (0.116–0.355) |
| Model2 → Model 3a (TyG) | 0.687 | 0.727 | 0.041 | <0.001 | 0.051 (0.037–0.064) | 0.400 (0.285–0.511) |
| Model2 → Model 3b (TyG-WWI) | 0.687 | 0.794 | 0.108 | <0.001 | 0.150 (0.129–0.172) | 0.695 (0.582–0.806) |
| Model2 → Model 3c (TyG-CVAI) | 0.687 | 0.738 | 0.051 | <0.001 | 0.065 (0.050–0.081) | 0.485 (0.373–0.597) |
| Model2 → Model 3d (TyG-BRI) | 0.687 | 0.719 | 0.032 | <0.001 | 0.039 (0.027–0.051) | 0.408 (0.295–0.529) |

**Table S2.** Discriminatory performance of different nested models was compared based on out-of-fold predictions, including AUC, ΔAUC (DeLong’s test), Integrated Discrimination Improvement (IDI), and continuous Net Reclassification Index (NRI). Model 1 is the traditional risk factor model; Model 2 includes Model 1 plus original scale AHI; Models 3a–3d further include Z-score standardized TyG, TyG-WWI, TyG-CVAI, and TyG-BRI, respectively. This table reflects the incremental gain of the models within a cross-validation framework, providing better control over internal optimism bias.

Figure S6: Calibration curves and Decision Curve Analysis (DCA) based on out-of-fold predictions


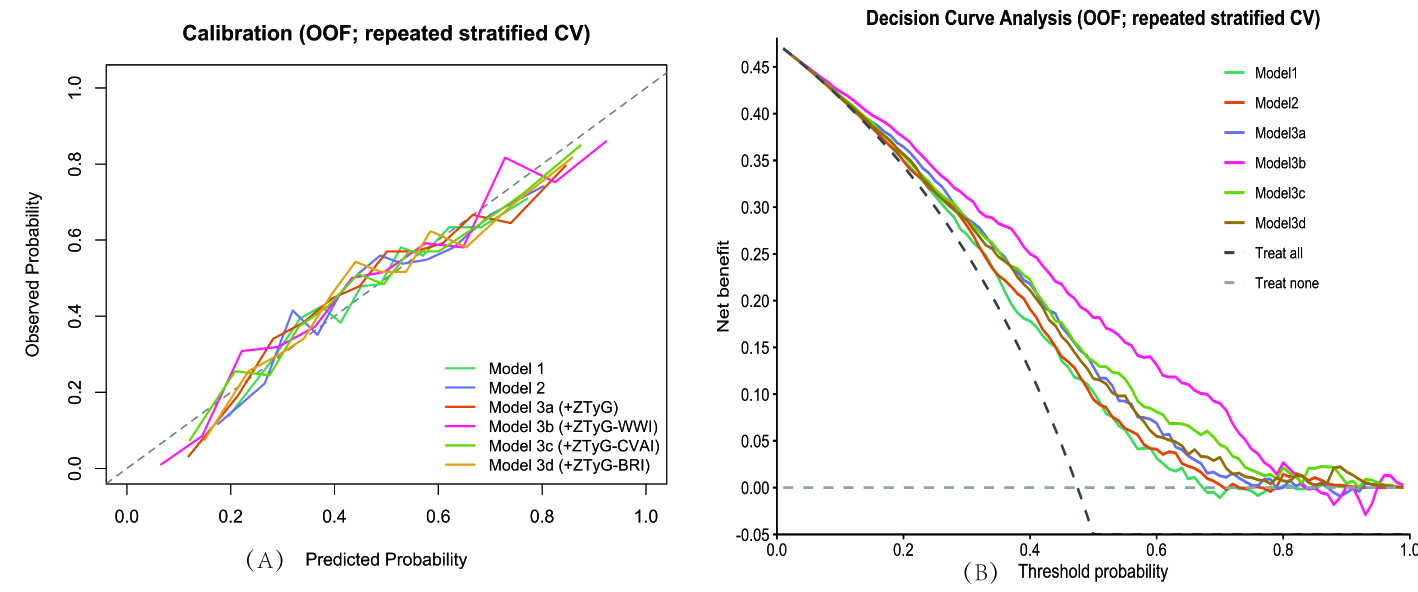


**Figure S6.** Candidate models were evaluated for calibration and clinical net benefit using out-of-fold predictions from repeated stratified 10-fold cross-validation.

Table S3: Event incidence and composition in the full follow-up population

| **Event component** | **Number of events** | **Total population** | **Event proportion, %** |
| --- | --- | --- | --- |
| Cardiac death | 11 | 1122 | 1.0 |
| Non-fatal myocardial infarction | 22 | 1122 | 2.0 |
| Non-fatal stroke | 17 | 1122 | 1.5 |
| Hospitalization for heart failure | 9 | 1122 | 0.8 |
| Malignant arrhythmia | 8 | 1122 | 0.7 |
| Unplanned revascularization | 13 | 1122 | 1.1 |
| **Overall MACE** | **80** | **1122** | **7.1** |

**Table S3.** This table details the overall occurrence of Major Adverse Cardiovascular Events (MACE) and the number and proportion of individual component endpoints during the follow-up period. Components include cardiac death, non-fatal myocardial infarction, non-fatal stroke, hospitalization for heart failure, malignant arrhythmia, and unplanned revascularization, describing the cardiovascular outcome burden in the study cohort.

Table S4：Association between TyG-related indices and Hard MACE risk: Cox proportional hazards model analysis

| **Exposure** | **Model 1**  **HR (95% CI)** | **P value** | **Model 2**  **HR (95% CI)** | **P value** | **Model 3**  **HR (95% CI)** | **P value** |
| --- | --- | --- | --- | --- | --- | --- |
| **TyG** | 1.926 (1.489–2.492) | <0.001 | 1.529 (1.148–2.036) | 0.004 | 1.444 (1.080–1.930) | 0.013 |
| **TyG-CVAI** | 1.563 (1.275–1.915) | <0.001 | 1.430 (1.108–1.846) | 0.006 | 1.328 (1.015–1.737) | 0.038 |
| **TyG-WWI** | 2.015 (1.624–2.501) | <0.001 | 1.801 (1.384–2.345) | <0.001 | 1.713 (1.303–2.251) | <0.001 |
| **TyG-BRI** | 1.369 (1.132–1.656) | 0.001 | 1.256 (1.001–1.575) | 0.049 | 1.185 (0.936–1.499) | 0.159 |

**Table S4.** Cox proportional hazards models were used to evaluate the association between Hard MACE risk and a 1-SD increase in Z-score standardized TyG, TyG-CVAI, TyG-WWI, and TyG-BRI. Model 1 is unadjusted; Model 2 is adjusted for age, sex, hypertension, diabetes, systolic blood pressure, smoking, LDL-C, and WBC; Model 3 further adjusts for AHI. Hard MACE is defined as the composite of cardiac death, non-fatal myocardial infarction, stroke, and unplanned revascularization.

Table S5: Cox regression analysis of TyG, TyG-WWI, TyG-BRI, and TyG-CVAI with MACE in the fully adjusted model

| **Index (Quartiles)** | **Q2 vs Q1 HR (95%CI)** | **P value** | **Q3 vs Q1 HR (95%CI)** | **P value** | **Q4 vs Q1 HR (95%CI)** | **P value** | **P for trend** |
| --- | --- | --- | --- | --- | --- | --- | --- |
| **TyG** | 0.945 (0.395–2.261) | 0.899 | 1.578 (0.717–3.473) | 0.258 | 1.410 (0.630–3.156) | 0.404 | 0.206 |
| **TyG-CVAI** | 1.439 (0.576–3.596) | 0.436 | 2.133 (0.886–5.134) | 0.091 | 2.798 (1.187–6.592) | 0.019 | 0.006 |
| **TyG-WWI** | 1.564 (0.622–3.938) | 0.342 | 1.541 (0.615–3.862) | 0.357 | 3.399 (1.473–7.846) | 0.004 | 0.001 |
| **TyG-BRI** | 1.109 (0.488–2.521) | 0.804 | 1.546 (0.723–3.308) | 0.262 | 2.176 (1.038–4.561) | 0.039 | 0.016 |

**Table S5.** In the fully adjusted Cox model, the associations between quartiles of Z-score standardized TyG, TyG-CVAI, TyG-WWI, and TyG-BRI and MACE risk were compared, using Q1 as the reference group. Hazard ratios (HR), 95% CIs, and P-values are reported for Q2, Q3, and Q4 relative to Q1. P for trend is provided to test for a dose-response relationship with increasing exposure.

Table S6: Assessment of Cox model complexity and events per variable (EPV）

| **Model** | **Number of covariates** | **Total parameters (exposure + covariates)** | **Number of events (MACE = 1)** | EPV |
| --- | --- | --- | --- | --- |
| Model 1 | 0 | 1 | 80 | 80.0 |
| Model 2 | 8 | 9 | 80 | 8.89 |
| Model 3 | 9 | 10 | 80 | 8.0 |

**Table S6.** This table lists the number of covariates, total parameters, and the corresponding events per variable (EPV) for different Cox models to assess model complexity and parameter stability. Model 2 covariates include age, sex, hypertension, diabetes, SBP, smoking, LDL-C, and WBC; Model 3 adds original scale AHI to Model 2. Since lower EPV may increase the risk of overfitting, this study further utilized Ridge Cox models for sensitivity analysis.

Table S7: Comparison of association estimates for TyG-related indices and MACE between standard and Ridge Cox models

| **Exposure (per 1-SD increase)** | **Standard Cox HR (95% CI)** | **Standard Cox P value** | λ_min_ | Ridge HR（λ_min_） | **HR range (λ = 0–10)** |
| --- | --- | --- | --- | --- | --- |
| TyG | 1.251（0.971–1.611） | 0.083 | 0.622 | 1.05 | 1.004–1.250 |
| TyG-CVAI | 1.284（1.017–1.621） | 0.036 | 2.51 | 1.02 | 1.004–1.281 |
| TyG-WWI | 1.543（1.216–1.959） | <0.001 | 4.35 | 1.01 | 1.005–1.536 |
| TyG-BRI | 1.185（0.966–1.453） | 0.103 | 0.516 | 1.05 | 1.003–1.184 |

**Table S7.** Estimates for the association between a 1-SD increase in TyG-related indices and MACE risk were compared between the standard Cox model (Model 3) and the Ridge Cox model. The Ridge Cox model utilized event-stratified 5-fold cross-validation to select the optimal penalty parameter (λ_min)_. Fitting was performed across a pre-specified range (λ=0–10). The corresponding Ridge HRs and HR ranges are reported to evaluate the stability of effect estimates across varying regularization strengths.

Figure S7: Time-dependent ROC curves for different Cox models predicting MACE during follow-up


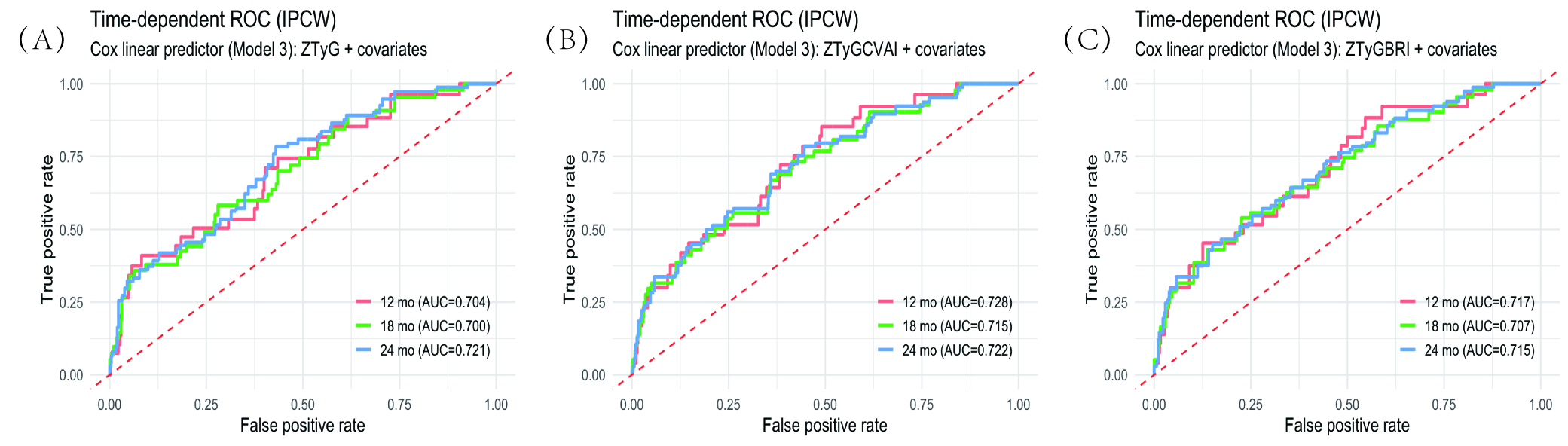


**Figure S7.** Time-dependent ROC curves were used to compare the dynamic discriminatory performance of different Cox models for MACE at pre-specified follow-up time points (12, 18, and 24 months). The AUC for each candidate model at different time points is displayed to reflect the stability of predictive performance over time.

Figure S8: Sensitivity analysis of exploratory statistical decomposition


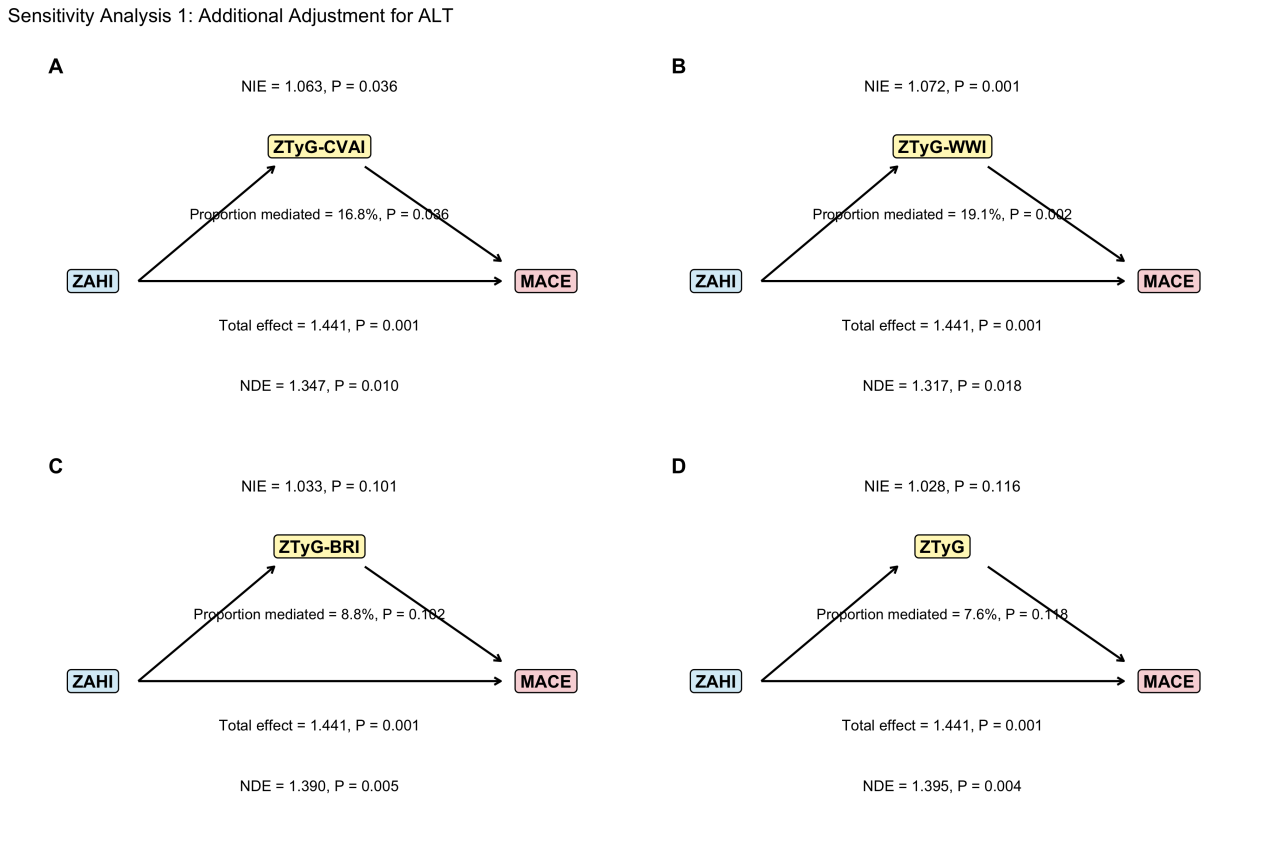


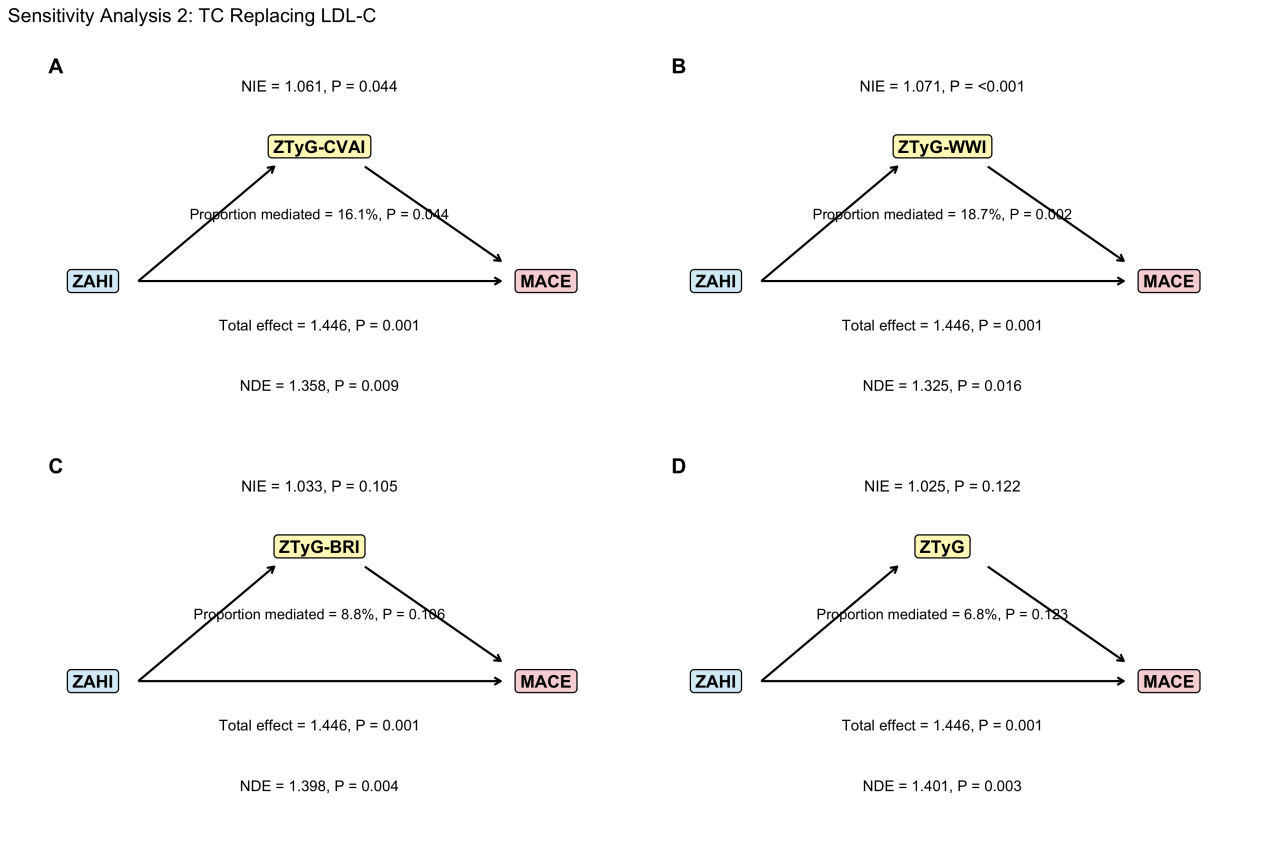


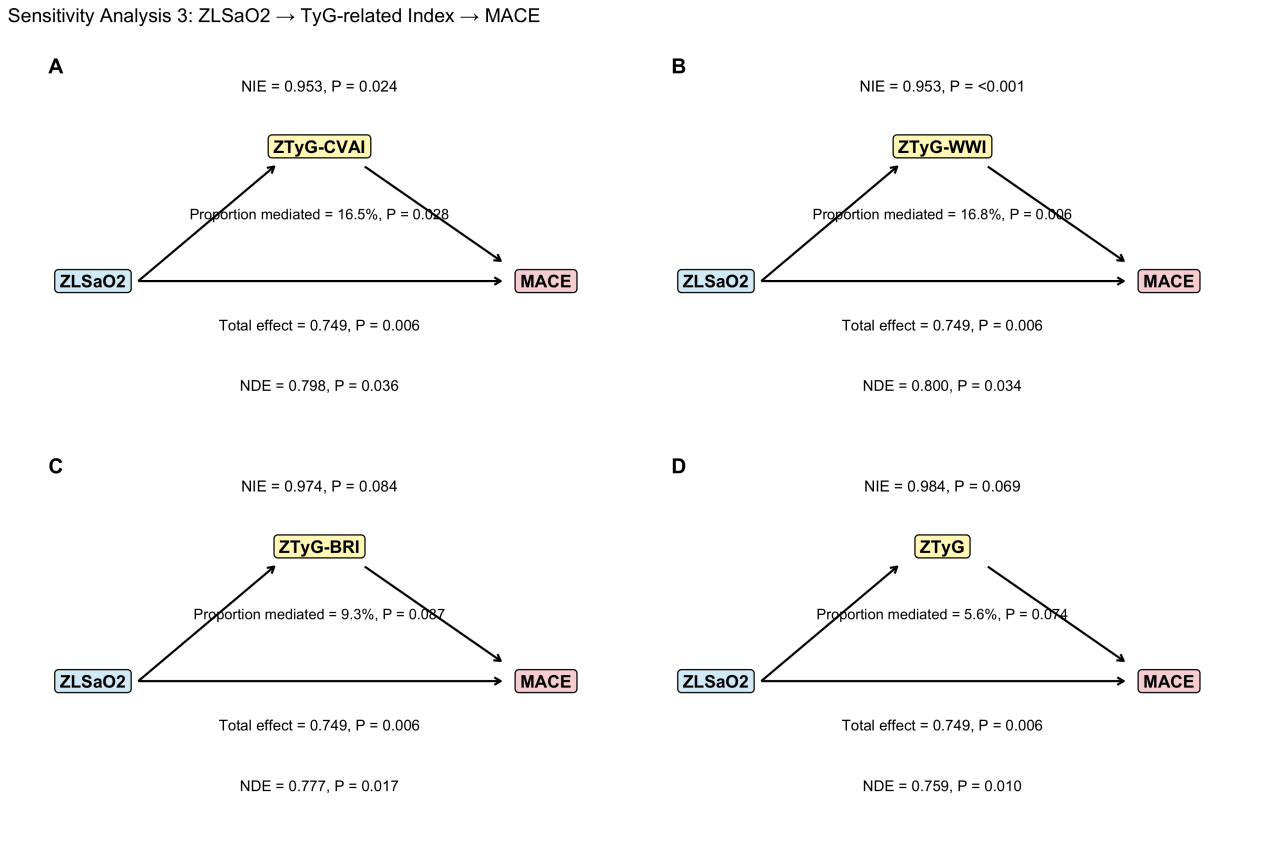


**Figure S8.** Multiple pre-specified sensitivity analyses were conducted on the results of the exploratory statistical decomposition, including: additional adjustment for ALT in the fully adjusted model, replacement of LDL-C with TC, and repeated modeling replacing Z-score standardized AHI with Z-score standardized LSaO₂ or MSaO₂. The figure shows the effect estimates and 95% CIs for the Total Effect (TE), Natural Direct Effect (NDE), and Natural Indirect Effect (NIE) under different model settings to assess the robustness of the indirect pathway signals.
